# Mass drug administration for the acceleration of malaria elimination in a region of Myanmar with artemisinin-resistant falciparum malaria: a cluster-randomised trial

**DOI:** 10.1016/S1473-3099(20)30997-X

**Published:** 2021-06-18

**Authors:** Alistair R D McLean, Chanida Indrasuta, Zay Soe Khant, Aung Kyaw Phyo, Sai Maung Maung, James Heaton, Hein Aung, Ye Aung, Kyaw Soe, Myo Maung Maung Swe, Lorenz von Seidlein, Ni Ni Tun, Kyaw Myo Tun, Nicholas P J Day, Elizabeth A Ashley, Thaung Hlaing, Thar Tun Kyaw, Arjen M Dondorp, Mallika Imwong, Nicholas J White, Frank M Smithuis

**Affiliations:** 1 Medical Action Myanmar, Yangon, Myanmar; Myanmar Oxford Clinical Research Unit, Yangon, Myanmar; Centre for Tropical Medicine and Global Health, Nuffield Department of Clinical Medicine, University of Oxford, Oxford, UK; 2 Medical Action Myanmar, Yangon, Myanmar; 3 Myanmar Oxford Clinical Research Unit, Yangon, Myanmar; 4 Mahidol-Oxford Tropical Medicine Research Unit, Faculty of Tropical Medicine, Mahidol University, Bangkok, Thailand; 5 Medical Action Myanmar, Yangon, Myanmar; Myanmar Oxford Clinical Research Unit, Yangon, Myanmar; 6 Department of Preventive and Social Medicine, Defence Services Medical Academy, Yangon, Myanmar; 7 Centre for Tropical Medicine and Global Health, Nuffield Department of Clinical Medicine, University of Oxford, Oxford, UK; Mahidol-Oxford Tropical Medicine Research Unit, Faculty of Tropical Medicine, Mahidol University, Bangkok, Thailand; 8 Myanmar Oxford Clinical Research Unit, Yangon, Myanmar; Centre for Tropical Medicine and Global Health, Nuffield Department of Clinical Medicine, University of Oxford, Oxford, UK; 9 Department of Public Health, Ministry of Health and Sports, Nay Pyi Taw, Myanmar; 10 Centre for Tropical Medicine and Global Health, Nuffield Department of Clinical Medicine, University of Oxford, Oxford, UK; Mahidol-Oxford Tropical Medicine Research Unit, Faculty of Tropical Medicine, Mahidol University, Bangkok, Thailand; Department of Molecular Tropical Medicine and Genetics, Faculty of Tropical Medicine, Mahidol University, Bangkok, Thailand; 11 Medical Action Myanmar, Yangon, Myanmar; Myanmar Oxford Clinical Research Unit, Yangon, Myanmar; Centre for Tropical Medicine and Global Health, Nuffield Department of Clinical Medicine, University of Oxford, Oxford, UK

## Abstract

**Background:**

To contain multidrug-resistant Plasmodium falciparum, malaria elimination in the Greater Mekong subregion needs to be accelerated while current antimalarials remain effective. We evaluated the safety, effectiveness, and potential resistance selection of dihydroartemisinin-piperaquine mass drug administration (MDA) in a region with artemisinin resistance in Myanmar.

**Methods:**

We did a cluster-randomised controlled trial in rural community clusters in Kayin (Karen) state in southeast Myanmar. Malaria prevalence was assessed using ultrasensitive quantitative PCR (uPCR) in villages that were operationally suitable for MDA (villages with community willingness, no other malaria control campaigns, and a population of 50-1200). Villages were eligible to participate if the prevalence of malaria (all species) in adults was greater than 30% or P falciparum prevalence was greater than 10% (or both). Contiguous villages were combined into clusters. Eligible clusters were paired based on P falciparum prevalence (estimates within 10%) and proximity. Community health workers provided routine malaria case management and distributed long-lasting insecticidal bed-nets (LLINs) in all clusters. Randomisation of clusters (1:1) to the MDA intervention group or control group was by public coin-flip. Group allocations were not concealed. Three MDA rounds (3 days of supervised dihydroartemisinin-piperaquine [target total dose 7 mg/kg dihydroartemisinin and 55 mg/kg piperaquine] and single low-dose primaquine [target dose 0·25 mg base per kg]) were delivered to intervention clusters. Parasitaemia prevalence was assessed at 3, 5, 10, 15, 21, 27, and 33 months. The primary outcomes were P falciparum prevalence at months 3 and 10. All clusters were included in the primary analysis. Adverse events were monitored from the first MDA dose until 1 month after the final dose, or until resolution of any adverse event occurring during follow-up. This trial is registered with ClinicalTrials.gov, NCT01872702.

**Findings:**

Baseline uPCR malaria surveys were done in January, 2015, in 43 villages that were operationally suitable for MDA (2671 individuals). 18 villages met the eligibility criteria. Three villages in close proximity were combined into one cluster because a border between them could not be defined. This gave a total of 16 clusters in eight pairs. In the intervention clusters, MDA was delivered from March 4 to March 17, from March 30 to April 10, and from April 27 to May 10, 2015. The weighted mean absolute difference in P falciparum prevalence in the MDA group relative to the control group was -10·6% (95% CI -15·1 to -6·1; p=0·0008) at month 3 and -4·5% (-10·9 to 1·9; p=0·14) at month 10. At month 3, the weighted P falciparum prevalence was 1·4% (0·6 to 3·6; 12 of 747) in the MDA group and 10·6% (7·0 to 15·6; 56 of 485) in the control group. Corresponding prevalences at month 10 were 3·2% (1·5 to 6·8; 34 of 1013) and 5·8% (2·5 to 12·9; 33 of 515). Adverse events were reported for 151 (3·6%) of 4173 treated individuals. The most common adverse events were dizziness (n=109) and rash or itching (n=20). No treatment-related deaths occurred.

**Interpretation:**

In this low-transmission setting, the substantial reduction in P falciparum prevalence resulting from support of community case management was accelerated by MDA. In addition to supporting community health worker case management and LLIN distribution, malaria elimination programmes should consider using MDA to reduce P falciparum prevalence rapidly in foci of higher transmission.

**Funding:**

The Global Fund to Fight AIDS, Tuberculosis and Malaria.

## Background

Substantial reductions in malaria morbidity and mortality have been observed over the past 15 years where highly effective artemisinin-based combination therapies (ACTs) and vector control measures have been deployed. In the Greater Mekong sub-region (GMS), where forest and forest fringe malaria transmission predominates, and conventional vector control interventions (insecticide treated mosquito nets and residual spraying) are less effective, the increased deployment of ACTs is likely to have contributed decisively to the 50% reduction in malaria cases between the years 2000-2014 ^1^. However, the emergence and spread of artemisinin resistant *P. falciparum* malaria in the region ^2–4^ and the subsequent loss of partner drugs ^5–10^ pose a major threat to the control and elimination of *P. falciparum* malaria. In Cambodia, Thailand, Laos and Vietnam artemisinin resistance was followed by partner drug resistance. Fitter multidrug-resistant parasites have spread across the Eastern GMS causing high ACT treatment failure rates. In Myanmar, which bears the majority of the malaria burden in the GMS, molecular markers associated with artemisinin resistance have also been found in many parts of the country ^11^ but artemether-lumefantrine, the first line ACT, has remained highly effective so far ^12^. Although artemisinin resistance in Myanmar has a different genetic origin to that in the Eastern GMS, widespread artemisinin resistance places selective pressure on the partner antimalarial (lumefantrine) threatening malaria control. In the past Myanmar was the likely gateway for the spread of first chloroquine and then sulfadoxine-pyrimethamine resistance in *P. falciparum* parasites from the eastern GMS to India, and then on to Africa. This cost millions of lives. Preventing the further spread of ACT resistance is a high priority and, as elimination of artemisinin resistance requires elimination of all falciparum malaria, all countries in the GMS have committed to malaria elimination by 2030 ^13^.

Early diagnosis and treatment with effective antimalarial medicines reduces symptomatic malaria ^14^ and it reduces malaria deaths but it does not necessarily eliminate the disease. Using ultra-sensitive quantitative PCR methods (uPCR) for the detection of low parasite densities has shown that even in low transmission settings, the majority of people infected with malaria parasites are asymptomatic and therefore do not seek treatment ^15^. Most of these infected individuals have low parasitaemias, below the detection threshold of routine testing methods ^16, 17^. These parasitaemias can persist for many months ^16, 17^ and, left untreated, fluctuate intermittently producing transmissible densities of gametocytes ^18–20^. This explains how malaria is sustained during the dry season each year when vector densities are very low.

Mass drug administration (MDA) targets this reservoir of malaria parasites. MDA aims to treat all people able to receive antimalarial drugs in a community regardless of their symptoms. The treatment clears the asymptomatic infections and prevents reinfection for the period that the slowly eliminated partner drug provides post treatment prophylaxis. The objective is to reduce malaria prevalence and transmission rapidly. Over the past century MDA has been used in many situations; sometimes resulting in sustained malaria elimination ^21–24^, whereas in others the impact has been transient. The popularity of MDA decreased with concerns over operational feasibility, long term effectiveness and the potential, under some circumstances, to increase drug resistance. Despite this, calls for urgent malaria elimination before artemisinin resistance spreads outside the GMS and a better understanding of the risks and benefits have renewed interest in MDA as an elimination accelerator. As people with asymptomatic parasitaemia have already controlled their infections, therapeutic responses are better than in symptomatic malaria. As with intermittent preventive treatments in higher transmission settings efficacy is greater than in the treatment of symptomatic malaria. Provided that MDA reduces the number of symptomatic malaria cases, then effective MDA with high coverage can reduce the emergence and spread of antimalarial resistance rather than increase it ^25^.

Dihydroartemisinin-piperaquine (DP) is a safe and well tolerated antimalarial ^26^. Although efficacy has declined markedly in the Eastern GMS it remains a very effective treatment for uncomplicated malaria in Myanmar and elsewhere ^26, 27^. DP provides a long period of post-treatment prophylaxis and can be used safely over multiple rounds ^28–30^. Primaquine (PQ) is a potent gametocytocide that has very low risk of toxicity when used at the recommended single low dose of 0.25mg/kg ^31, 32^. We performed a cluster-randomised controlled trial to assess the operational effectiveness and impact of MDA with DP and PQ in rural communities with a high prevalence of asymptomatic malaria carriage (“hotspots”) in a region with artemisinin resistance in Southeast Myanmar. After providing routine CHW community case management and distributing long-lasting insecticidal nets (LLIN) we assessed MDA safety and its impact on *P. falciparum* malaria incidence, prevalence, and on artemisinin and piperaquine resistance markers in a cluster randomised comparison.

## Methods

### Study area

**Figure 1:**
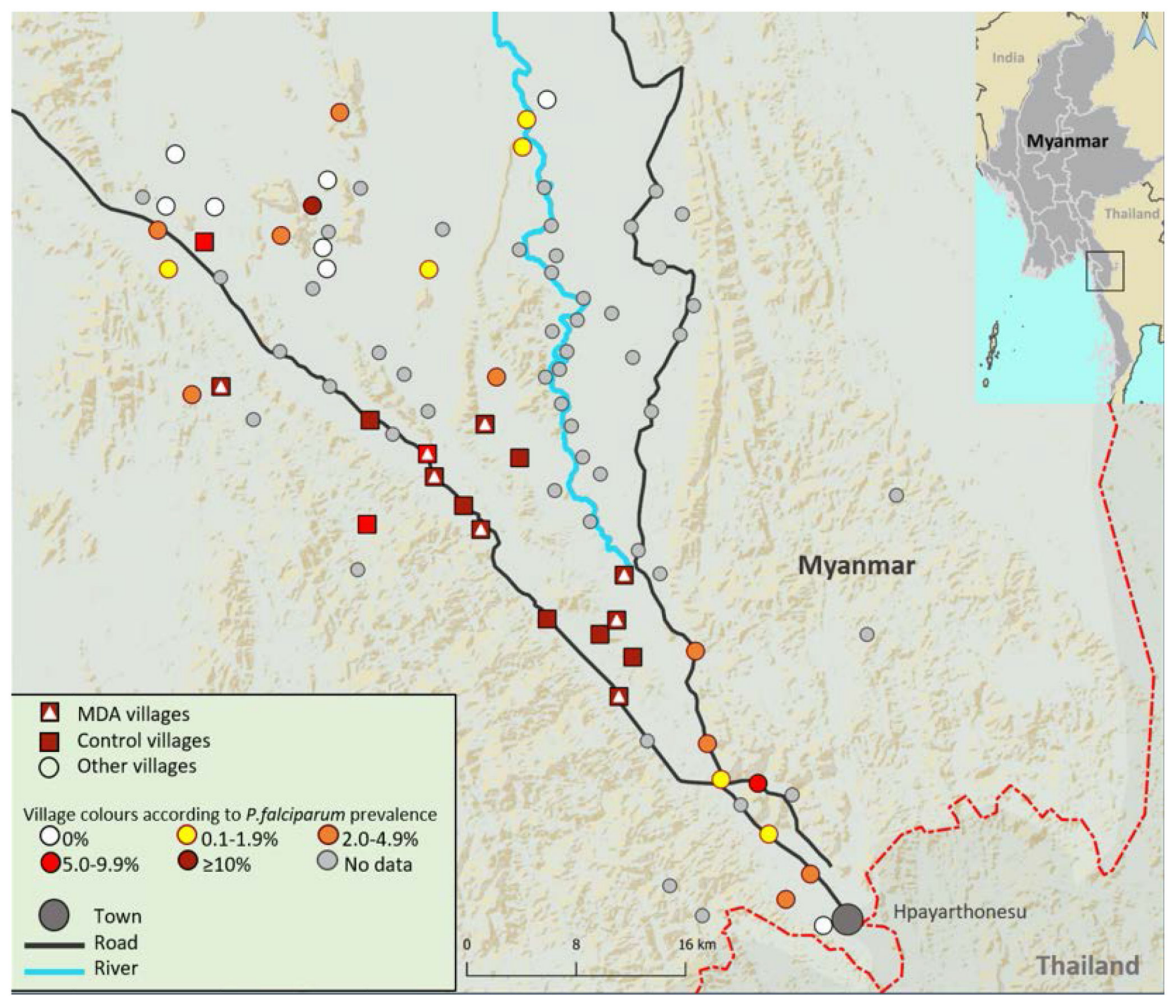
Map of study region in Eastern Kayin state with *P. falciparum* prevalences found at screening survey.

The study was conducted in Southern Kayin (Karen) state, in East Myanmar near the Myanmar-Thai border (Figure 1). The terrain varies from forested hills to flat areas with rice fields, scrub land and rivers. Yearly rainfall is high (3,000 – 4,800 mm/year). Malaria transmission is generally low and uneven with foci (“hot spots”) of higher intensity. Transmission occurs throughout the year with a nadir in the spring dry season and a peak during, and immediately after, the six months rainy season. Artemisinin resistant *P. falciparum* is prevalent in this area; a recent survey found kelch13 mutations in 46% of *P. falciparum* infections ^12^. Most of the population are Kayin (Karen) farmers. Villages are generally remote and small, with an average of approximately 600 people. The infrastructure is poor and access to formal health care is limited. In 2012, *Medical Action Myanmar* (MAM) instituted a network of community health workers (CHWs) in remote communities in this region to provide community-based malaria case management, in cooperation with the Myanmar National Malaria Control Program, as described previously ^14^.

### Baseline prevalence survey and cluster selection

We obtained a list of 58 villages from the Kayin state health authorities in a 1600 sq km area (Figure 1). In each village a local person was trained as a CHW and villagers were encouraged to consult the CHW in case of fever. Villages were assessed for their operational suitability to implement MDA. This was assessed as: village leadership and community willing to participate; no other organisations supporting malaria management; and village population between 50 and 1200. Forty-three villages fitted these criteria and were included in a baseline malaria prevalence survey conducted between 5 and 21 January 2015 using a validated uPCR with a lower limit of quantification of 22 parasites/mL ^15^. Three to four field teams each started at a house in a different corner of the village, and visited every fourth household. If that household was empty the team moved to the nearest house backward or forwards. From each selected household, a minimum of two persons over 18 years of age were invited to participate. After written informed consent was obtained, participants were asked questions about potential malaria symptoms, allergies and recent travel history, and a venous blood sample was taken and tested for malaria (all species) using a Rapid Diagnostic Test (RDT) (SD Bioline). This RDT detects *P. falciparum* HRP2 and plasmodium lactate dehydrogenase. In total 2,647 participants were sampled. Participants with a positive RDT were treated immediately according to national guidelines. Blood samples were transported to a field laboratory, where they were processed and stored at -20°C for a maximum of two weeks before transfer on dry ice to Bangkok, Thailand, for uPCR analysis (described previously ^15^). If there was sufficient DNA the propeller domain of *kelch13* (k13), a marker of artemisinin resistance, was sequenced and the *Pfplasmepsin2* copy number (a marker of piperaquine resistance) was quantified using RT-PCR on all *P. falciparum* positive samples.

Villages were eligible to participate in the MDA study if they fulfilled the following criteria:

1. The survey baseline malaria prevalence exceeded 30% and/or the *P. falciparum* malaria prevalence exceeded 10% in the adult population tested by uPCR. 2.
2. They were discrete geographically.
3. If the cluster could be paired to another cluster nearby with similar *P. falciparum* prevalence.

**Figure 2:**
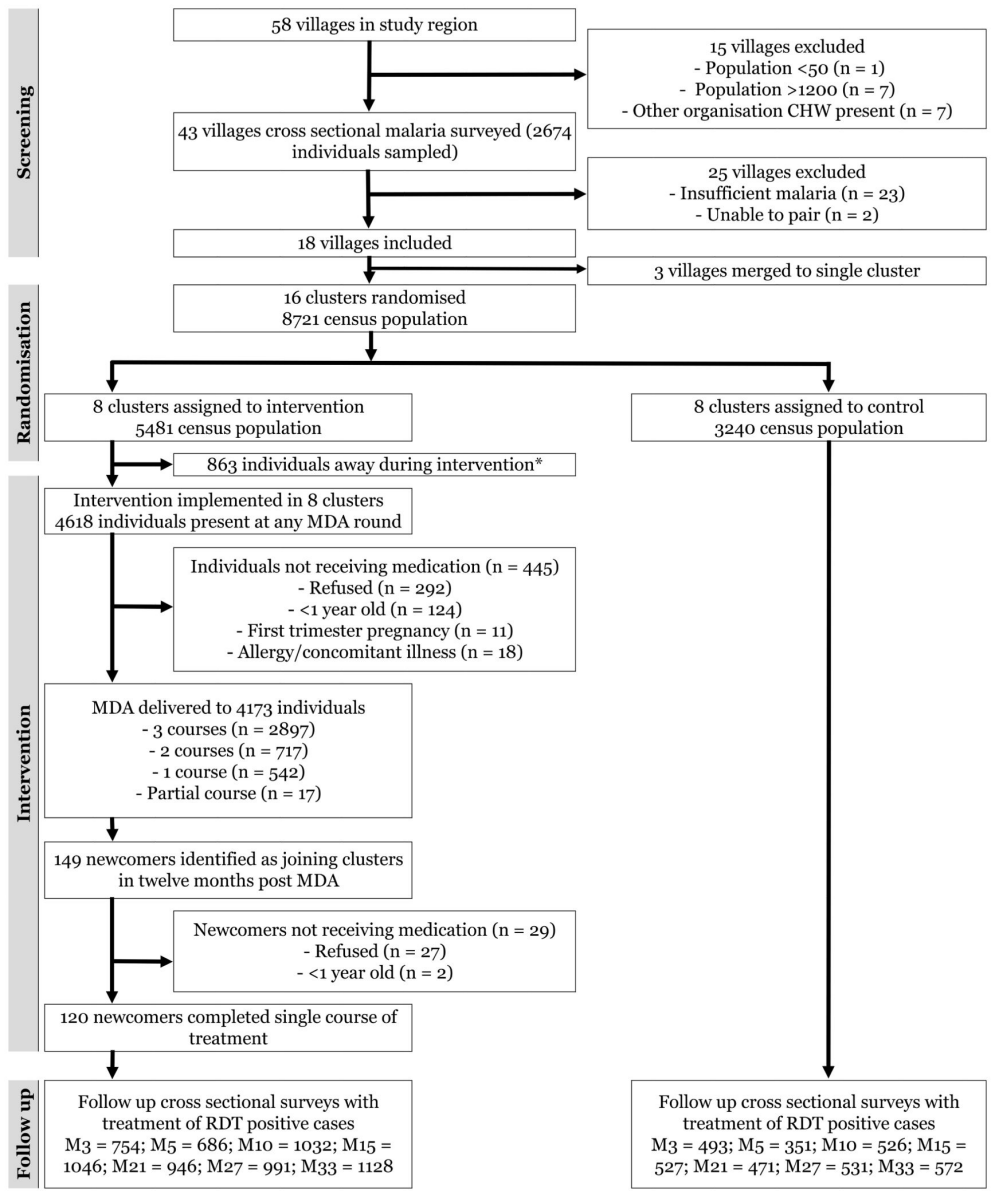
Study profile. MDA = Mass Drug Administration; RDT = Malaria Rapid Diagnostic Test; M=month i.e. M3, M33 = Month 3, Month 33; * Individuals present during census but away from the cluster for all rounds of medication delivery.

Eighteen villages were deemed eligible to participate in the study (Figure 2). In one case three small villages were combined into one cluster as they were in close proximity and a border between them could not be defined clearly. The sixteen clusters were matched in pairs based on: a) P. *falciparum* prevalence (estimates within 10%); b) proximity to each other; and c) distance to the main road. Matching was done in order to minimise confounding by environmental differences, likely to influence vector populations, malaria transmission intensities and population mobility. For each pair, one cluster was allocated randomly to the intervention arm by coin toss.

### Community engagement

Community leaders were first informed about the study and if they agreed study staff subsequently interacted with the communities in a series of community engagement activities described elsewhere ^33^. Community engagement was undertaken before each MDA round and uPCR survey. If the community agreed to participate, house visits were conducted to allow community members to discuss issues directly with the study team, to collect census data, to invite eligible participants, and to take participants’ informed consent. At each interaction it was reiterated that individuals could choose to participate or opt out at any time, regardless of the community decision.

### Diagnosis and treatment of symptomatic malaria

Patients presenting to the community health worker with signs or symptoms of malaria were tested using a rapid diagnostic test (SD Bioline). If the test was positive the patients was treated according to National guidelines with a standard regimen of artemether-lumefantrine (*P. falciparum*) or chloroquine plus the radical curative regimen of primaquine nationally recommended at the time; 0.75mg/kg once weekly for 8 weeks (*P. vivax*).

### Mass antimalarial drug administration

**Figure 3:**
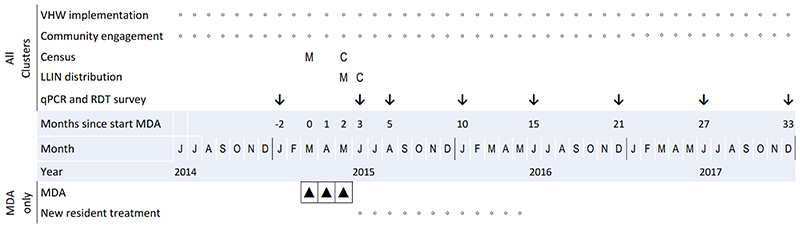
Study Timeline. Small circles denote ongoing activities; vertical arrows denote activities at specific times; M and C denote activities at specific times in MDA (M) and Control (C) clusters and black triangles represent a round of MDA.

MDA was delivered to eight intervention clusters, as three consecutive monthly distributions in March, April and May 2015, when malaria transmission was assumed to be at its lowest (Figure 3). Each drug distribution consisted of a three day course of dihydroartemisinin-piperaquine (DP) (Eurartesim®, Sigma-Tau; target total dose of 7 mg/kg dihydroartemisinin and 55 mg/kg piperaquine) and a single dose of primaquine (PQ) (Remedica; target dose 0.25mg base/kg) on the first day. MDA was offered to all eligible community members present during MDA distribution. Participants provided written informed consent on the first day and verbal consent on subsequent treatment days. Participants completed symptom and adverse event questionnaires. Pregnant women in the first trimester, children under one year old, and individuals with a previous adverse reaction to study medication or experiencing active acute concurrent illness were not included. Pregnant or lactating women were not given primaquine.

All drug administrations were observed. MDA delivery lasted five days per cluster to ensure all community members were offered medication and were then monitored until the antimalarial course was completed. Participants who did not present to the distribution post were followed up in their homes. All villagers were informed that team members would remain in the community during the days following MDA to manage any adverse events. LLINs were distributed, one for every two people in every household, during the final round of MDA in May 2015 in the intervention clusters, and during the first follow up uPCR survey in June 2015 in the control clusters. After MDA, individuals with *P. falciparum* detected by RDT in intervention clusters underwent contact tracing whereby family members, neighbours within 50m of the patient’s residence and people who were with the patient in the suspected transmission area were offered RDT testing. During the 12 months after MDA, CHWs in intervention clusters offered a single course of DP + PQ to all new residents, defined as persons who arrived in the village with the intention of staying over 1 month. CHWs were instructed to monitor for new arrivals and field teams checked regularly with the village leader to identify new residents.

### Adverse event monitoring

The reporting period for adverse events (AEs) was from the day of administration of the first dose of study drugs until one month after final dose or until resolution of any AE that had occurred during the follow-up period. Participants were screened for AEs on each day of drug administration using open and closed questions designed to detect relevant AEs (dark urine, rash or itching, dizziness). Some serious adverse events were reported on other days or identified during home visits to participants. All SAEs were to be assessed independently by the study medical safety monitor and reported to the ERB of the Department of Medical Research in Myanmar.

### Cross sectional malaria prevalence surveys after MDA

Cross-sectional prevalence surveys were conducted in adults (children were excluded to comply with ethical committee requirements), in intervention and control villages 3, 5, 10, 15, 21, 27 and 33 months after the beginning of MDA. Field teams attempted to sample the same individuals where possible to allow for longitudinal analysis. If they were unavailable (usually because they were working in the fields or forest), another adult participant from the same household (or, if unavailable, from a neighbouring household) was selected based on similar characteristics (matched for sex, age within ten years, and occupation). As in the baseline prevalence survey, consent was obtained, RDTs were performed and venous blood was collected for uPCR analysis, *kelch13* sequencing and *Pfplasmepsin2* copy number quantification.

### Molecular genotyping of *P. falciparum* resistance markers

Polymorphism in the *PfKelch* gene was examined by nested PCR amplification covering the propeller region of the gene as described previously ^3^ followed by sequencing of the gene by ABI Sequencer (Macrogen Inc, South Korea). The sequencing results were then aligned against the *PfKelch* gene of reference strain 3D7 (putative PF13_0238 NCBI Reference Sequence (3D7): XM_001350122.1) and analysed with Bioedit software (Abbott, CA, USA). *Pfplasmepsin2/3* copy number was quantified using Relative quantitative Real-time PCR based on Taqman real time PCR on a Corbett Rotor-Gene™ Q (Corbett Research, Australia). Primers and probes have been described previously ^34^. Amplification was performed in triplicate on a total volume of 10 μL as multiplex PCR using Quantitec Multiplex PCR no ROX (QIAgen, Germany). Copy numbers were calculated using the formula: copy number =**2** ^-ΔΔct^; with ΔΔ **C**_t_ denoting the difference between Δ C_t_ of the unknown sample and Δ C_t_ of the reference sample. A cut-off copy number of 1.5 was used to define *Pfplasmepsin2/3* and *pfmdr1* amplification. Reactions were repeated whenever the profile did not conform to exponential kinetics, or ΔΔCt spread was >1.5, or the Ct value was >35. To confirm amplification and resolve indeterminate results, samples passing these criteria but with an estimated copy number >1.3 were also re-tested once, the second result counting as final.

### Analysis

The primary outcome of the study was the prevalence of uPCR detected *P. falciparum* infections in the intervention and control arms one month after completion of MDA (Month 3) and 12 months after the baseline survey. MDA coverage was defined as the number of people who received MDA as a proportion of the total population physically present in the cluster on any day of MDA distribution. Incident malaria cases were defined as persons who attended a CHW and tested as malaria positive by RDT. Field data were collected on paper reporting forms and entered afterwards into an OpenClinica (Waltham, USA) electronic database. For baseline malaria surveys, the sample size was calculated to estimate a 10% prevalence of *P. falciparum* with 6% precision, alpha 0.05 and 90% confidence after applying a finite population correction. Later surveys had increased sample sizes to increase the precision of the prevalence estimates.

For the randomised comparison the cluster was the unit of randomisation and the unit of analysis. Cross-sectional prevalence survey data and incidence data were aggregated at cluster level for analyses to account for the cluster randomised trial study design. We compared the prevalence and incidence of malaria in the MDA clusters to control clusters using a weighted paired *t* test ^35^. Incidence density of *P. falciparum* and *P. vivax* infections was defined as number of incident malaria cases per 1,000 person years at risk, using total cluster population at census to estimate total person years. Incidence was compared between arms for each year after MDA was administered. For seven months during which one of the MDA clusters had no active CHW, incidence from the paired control village was not included in the comparison. For calculation of the weighted prevalence of *P. falciparum* and *P. vivax* in the intervention and control arms at each time point, clusters were weighted by the reciprocal of the sampling fraction with application of a finite population correction using the total adult population. Finite population corrections were also applied to the calculations of 95% confidence intervals for the cluster-specific prevalences. Where zero infections were observed in a cluster survey, exact/Clopper-Pearson binomial 95% confidence intervals were calculated.

For the exploratory analysis of individual-level factors associated with *P. falciparum* infection, multilevel mixed-effects logistic regression models were used with study cluster specified as a random effect. Exposures were sex, age (continuous, per 10 years), reported recent forest stay, reported recent overnight stay outside of the village, received MDA drugs, and presence in village during MDA administration. An estimate and 95% confidence interval of the unadjusted odds ratio was presented for each exposure variable.

For the post-hoc exploratory analysis of the incidence of new uPCR detected *P. falciparum* and *P. vivax* infections occurring after the intervention we assumed that positive uPCR infections detected within the same individual without an interim uPCR negative result were a single continuous infection ^36^. We assumed that all instances of a *P. falciparum* positive uPCR test that were preceded either by a negative uPCR test, or were the first uPCR test conducted in that individual were new infections. Individual exposure time for this analysis was defined as the number of days in the catchment area following the beginning of the MDA. We assumed an individual who was present at two consecutive qPCR surveys was present for the entirety of the time between the surveys. If a resident was missing during a qPCR survey we assumed they remained under exposure time for 45 days after their last survey and 45 days before the next survey they were present for ^37^. We assumed a newly tested individual began their exposure period 45 days before their first test. Data were analysed using Stata (version 14.2, StataCorp, College Station, Texas).

### Ethics approval and role of funding source

This study was approved by the Department of Health and ethical approval was given by the Ethics Review Committees of the Department of Medical Research in Myanmar and the Oxford Tropical Research Ethics Committee. The trial was registered with clinicaltrials.gov (id: NCT01872702). The funding source had no role in the study design, data collection, data analysis, data interpretation, or writing of the report. The corresponding author had full access to all the data in the study and had final responsibility for the decision to submit for publication.

## Results

### Baseline surveys and screening

**Figure 4:**
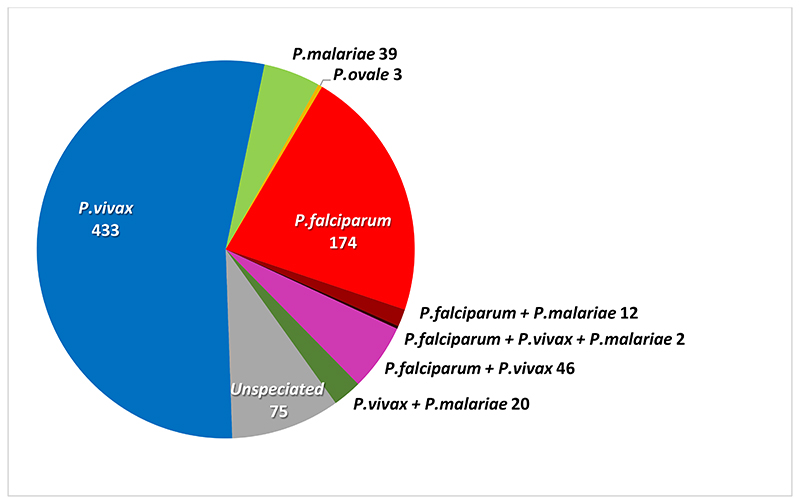
Malaria species detected by uPCR at baseline survey.

The baseline uPCR malaria surveys were performed in January 2015 on 2,674 individuals in 43 villages (14.9% of the total estimated population of 17,913) (Supplementary Table 1). Overall 804 (30%) persons tested positive for malaria; 234 (9%) for *P. falciparum*, 501 (19%) for *P. vivax*, 73 (2.7%) for *P. malariae*, 3 (0.1%) for *P. ovale*; and 75 (2.8%) for unspeciated malaria (insufficient DNA for speciation) (figure 4). *P. knowlesi* and *P. cynomolgi* were not detected. 80 persons had mixed infections of 2 (78) or 3 species (2).

During the census 8,721 people were present in the selected study clusters, 5,481 in the MDA clusters and 3,240 in the control clusters. The age and sex distributions were similar for the MDA and the control villages (Table 1). Baseline *P. falciparum* prevalences were similar in the intervention (15.9%) and control (16.9%) arms and the intra-cluster correlation coefficient was 0.056.

Malaria prevalence varied widely with median (minimum, 25^th^ percentile, 75^th^ percentile, maximum) village positivity rates of 4% (0%, 1%, 15%, 42%) for *P. falciparum* and 16% (1%, 11%, 29%, 40%) for *P. vivax* respectively. RDTs were positive in 30 (13%) of the 234 people who tested positive for *P. falciparum* by uPCR and 3 (0.6%) of the 501 people who tested positive for *P. vivax* by uPCR (p<0.0001). Of the individuals who tested positive for *P. falciparum* and *P. vivax* by RDT and uPCR 87% (26/30) and 67% (2/3) respectively did not have fever or a complaint of fever. RDTs were positive in 7 (0.3%) of the 2437 people negative for *P. falciparum* by uPCR and 3 (0.1%) of the 2170 people negative for *P. vivax* by uPCR. None of these RDT positive uPCR negative patients had fever or a history of fever.

The geometric mean estimated parasite density, based on the uPCR, of *P. falciparum* infections was 35,309 parasites/mL (95% CI: 10,478-60,881; 25th-75th percentiles: 2,159-557,908/mL). *P. vivax* infections had lower densities; geometric mean 5,935 parasites/mL (4,710-7,480; 25th-75th percentiles: 1,016-32,425/mL).

### MDA participation and coverage

Of the 5,481 people present in the MDA intervention villages at any time during the census or during the MDA delivery, 863 (15.7%) were present only at the census. This reflected substantial population mobility. The MDA cluster population varied markedly during the 3 MDA rounds with 4,000, 4,104, and 4,176 people present at months 0, 1 and 2 respectively. In total, 4618 individuals were seen across the three MDA rounds and 3,544 (76.7%) were present for all three rounds. MDA uptake was high; of 4,618 people present during the intervention period, 4,156 (90.0%) received at least one full course of treatment, 292 (6.3%) refused MDA while 153 (3.3%) were ineligible for medication because of age <1 year (124; 81%), pregnancy (11; 7%) concomitant illness (4; 3%) or previous suspected adverse reaction to the drugs (4; 3%) (Supplementary Table 2). Across all rounds 17 (0.4%) people started but did not complete a full course of medication (8 left the study area after the 1^st^ dose, 8 refused further medication because of side effects after the 1^st^ dose, and one became ineligible for medication during their course).

Acceptance of MDA among people present was 3,432/4,000 (85.8%), 3,542/4,104 (86.3%) and 3,647/4,176 (87.3%) for each MDA round respectively (Supplementary Table 3). One cluster had a lower coverage of 75% (337/450), as part of that community was of a different ethnic Karen group who prefer traditional medicine to ‘western medicines’. During the 12 months following the MDA, 149 new residents were identified in the intervention communities, of whom 120 (81%) took a course of MDA, 2 were ineligible (age <1 year) and 27 (18%) refused the treatment.

### Malaria prevalence

#### a) P. falciparum

*P. falciparum* prevalence estimated by uPCR declined rapidly after MDA from 14.5% (95% CI; 10.0%, 20.4%) at baseline to 1.4% (0.6%, 3.6%) at month 3, a 90% reduction (Figure 4A). All individuals who tested positive at baseline for *P. falciparum* (57) or *P. vivax* (93) and who took MDA drugs in the 3^rd^ round (month 2) and were tested subsequently at month 3, were negative. Twelve people had *P. falciparum* infections detected by uPCR at month 3, of whom eight had not received MDA. The other four individuals had received three full courses of MDA. Three had infections with K13 mutations indicating artemisinin resistance (two C580Y and one F446I). Thus among 626 individuals who were tested at month 3 after receiving a full course of DHA-piperaquine in month 2, 4 (0.6%) had a *P. falciparum* infection detected by uPCR.

**Figure 5:**
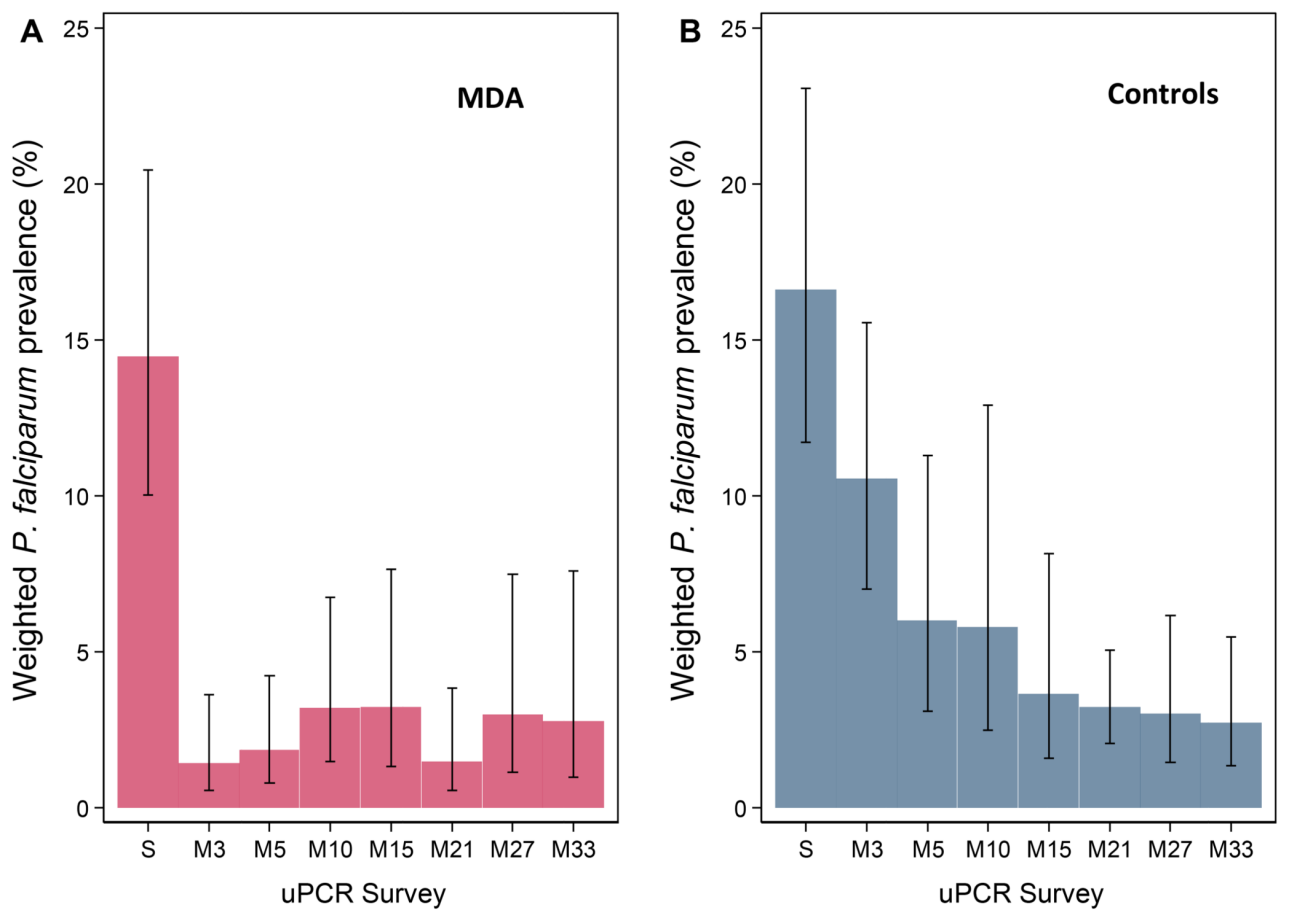
Weighted *P. falciparum* prevalence (assessed by uPCR) by arm. Weighted *P. falciparum* prevalence by survey time-points in MDA (A) and control (B) arms with 95% Confidence Intervals (capped bars). S = screening; M = month.

**Figure 6:**
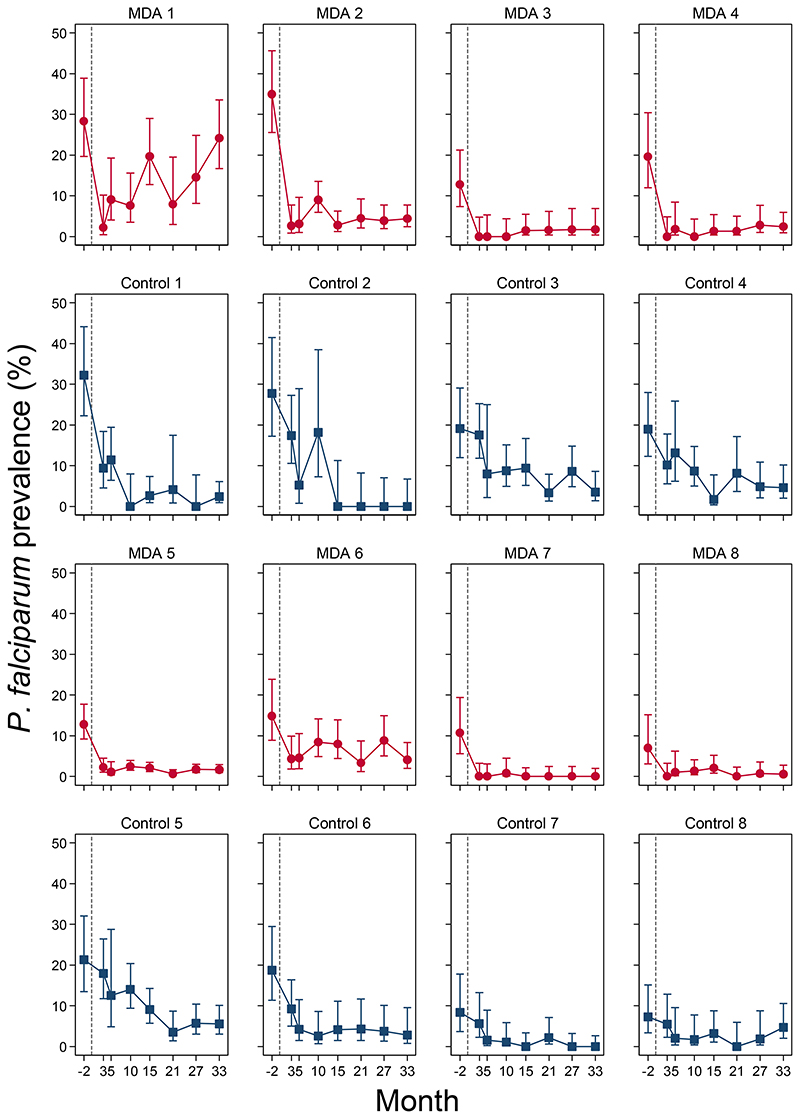
Individual *P. falciparum* prevalences (assessed by uPCR) by cluster pairs. A; Red circles (MDA clusters) and blue squares (control clusters) denote point estimates of *P. falciparum* prevalences, capped bars denote 95% confidence intervals.

*P. falciparum* prevalence in the MDA arm remained low in successive surveys, with estimated prevalences ranging from 1.5% to 3.2% (Figure 5A). At the final prevalence survey (month 33), the *P. falciparum* prevalence in the intervention arm was 2.8% (95% CI: 1.0%, 7.6%). Prevalence trends in most intervention clusters were consistent with the notable exception of MDA cluster 1, the only cluster without an active community health worker by the time of MDA administration. In this village there was an immediate reduction in prevalence after the MDA but a substantial rebound then followed (Figure 5). In the control villages *P. falciparum* prevalence also declined, but at a much slower rate; from 16.6% (95% CI; 11.7%, 23.1%) at baseline to 10.6% (7.0%, 15.6%) at month 3 and 2.7% (1.3%, 5.5%) at month 33 (Figure 5B). Prevalences in individual control clusters reflected the overall trends seen in the control arm (Figure 6).

**Figure 7:**
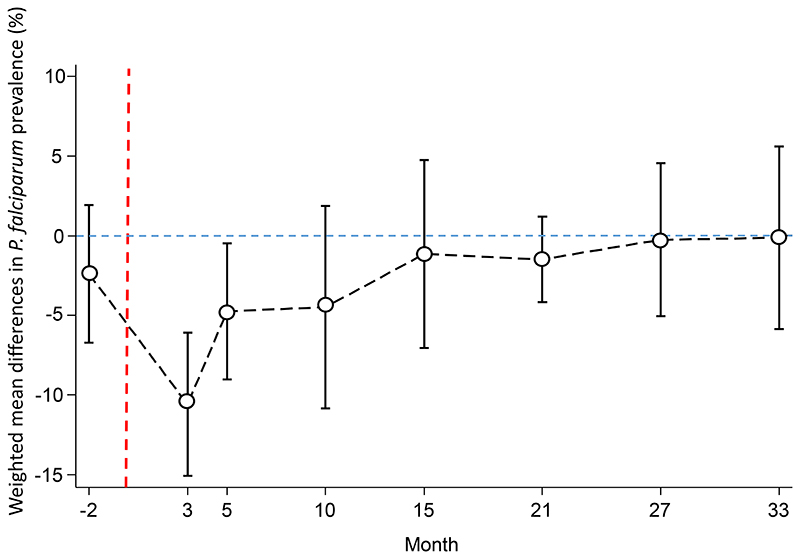
Weighted mean difference between the *P. falciparum* prevalence in the MDA and control arms. Circles denote point estimates and capped bars denote the 95% confidence intervals. Red dashed line denotes start of MDA.

At month 3, one month after MDA, clusters in the intervention arm had a significantly lower prevalence of uPCR detected *P. falciparum* infections than clusters in the control arm, with a weighted mean difference of -10.6% (95% CI; -15.1, -6.1%, p=0.001, Figure 7). The magnitude of the weighted mean difference between the study arms reduced over time, with a weighted mean difference of -4.8% (95% CI; -9.0%, -0.5%, p=0.03) at month 5; -4.5% (95% CI; -10.9, 1.9, p=0.14) at month 10; and -0.1% (95% CI; -5.9, 5.6, p=0.96) at month 33. RDTs identified 15% (77/528) of the uPCR detected *P. falciparum* infections in the intervention and control arms.

Exploratory analyses of individual level risk factors for uPCR *P. falciparum* positivity following the MDAs were conducted (Supplementary Table 4). Males were at greater odds of infection than females (Odds Ratio (OR) ranged from 5.5 (95% CI: 1.2, 25.6) at month 3 to 1.6 (0.8, 3.4) at month 27. There was no consistent association between age and asymptomatic *P. falciparum* infections.

Individuals who reported a recent (see Supplementary Table 5 for definitions of recent) overnight stay in the forest were at increased odds of *P. falciparum* infection. At month 3 the OR was 3.2 (95% CI: 0.9, 11.2) (3.5% *P. falciparum* prevalence in those reporting forest stays versus 0.9% in those not reporting forest stays). By month 21 the OR was 1.8 (95% CI: 0.6, 5.4) (2.1% versus 1.1%). By contrast there was no consistent association between reporting of a recent (Supplementary Table 5) non-forest overnight stay outside of the village and *P. falciparum* infection. There was also no consistent association between the reported frequency (Supplementary Table 6) of bednet use and detected *P. falciparum* infections.

Individuals who had not received MDA were more likely to have asymptomatic sub-microscopic *P. falciparum* infections than were MDA recipients. The differences were greatest immediately after MDA (OR = 19.0 (95% CI: 5.6, 64.7) (10% versus 0.6%) at month 3 than at later periods (Supplementary Table 4). Similarly, individuals who were not present in the village during MDA had more asymptomatic *P. falciparum* infections than those who were present. Again the differences were greater immediately after MDA (OR = 14.0 (95% CI: 4.3, 45.5)) at month 3 than at later periods (Supplementary Table 4).

After MDA 87 new *P. falciparum* infections were detected by uPCR in the control arm and 111 in the intervention arm. The incidence of new *P. falciparum* infections was 82.7 (95% CI: 66.3, 102.1) per 1000 person years in the control arm and 53.4 (43.9, 64.3) in the intervention arm, a weighted mean difference of -35.8 (95% CI: -121.8, 50.1; p=0.3571) new cases.

#### b) P. vivax

**Figure 8:**
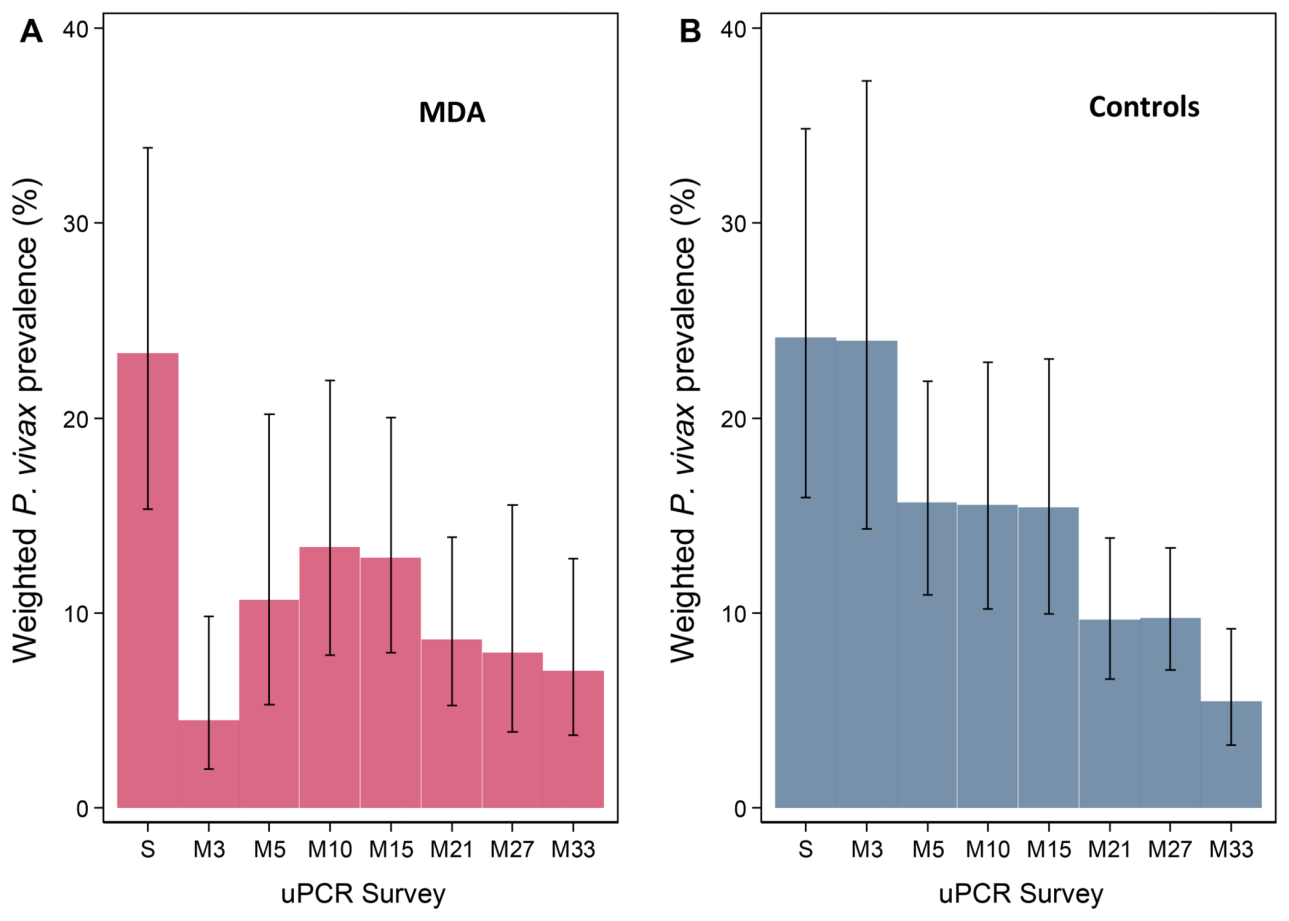
Weighted *P. vivax* prevalences (assessed by uPCR) by arms. Weighted *P. vivax* prevalences by survey time-points in MDA (A) and control (B) arms with 95% Confidence Intervals (capped bars). S = screening; M = month.

**Figure 9:**
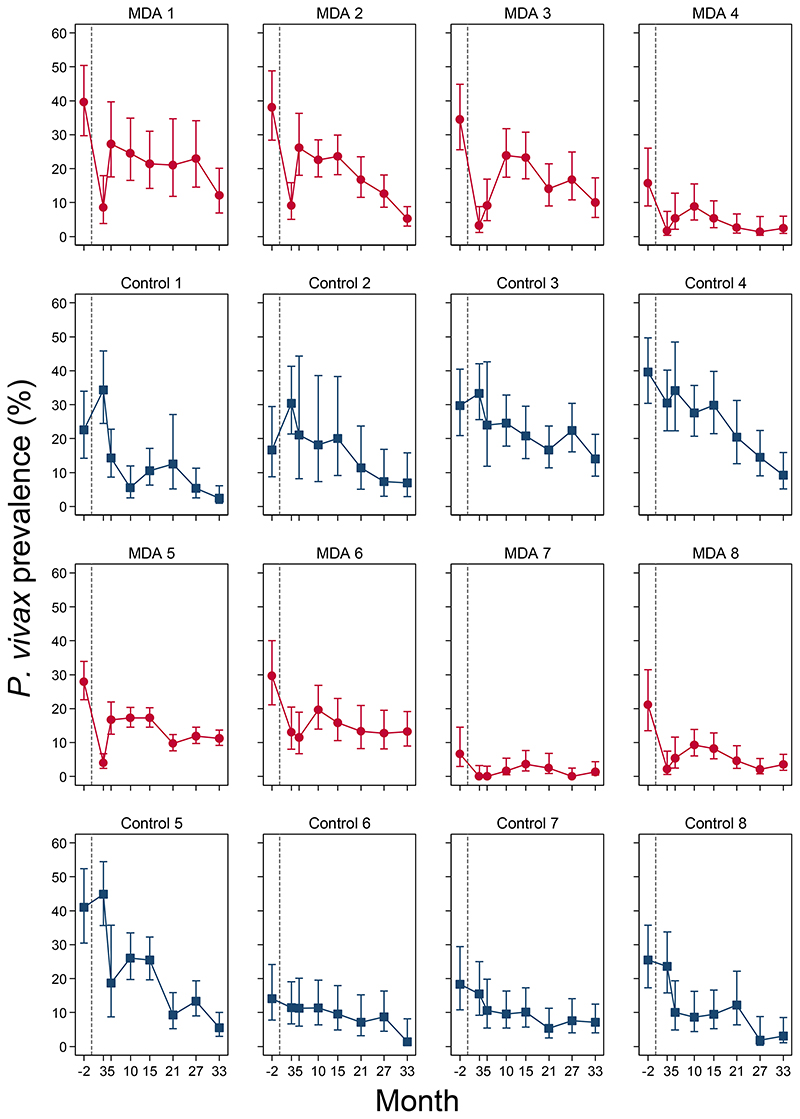
Individual *P. vivax* prevalences (assessed by uPCR) by cluster pairs. A; Red circles (MDA clusters) and blue squares (control clusters) denote point estimates of *P. vivax prevalence*, capped bars denote 95% confidence intervals.

*P. vivax* prevalence declined immediately following MDA in the intervention arm from 23.3% (95% CI; 15.3%, 33.9%) at baseline to 4.5% (2.0%, 9.8%) at month 3 (Figure 8A) but then increased to 13.4% (7.8%, 21.9%) at month 10 and thereafter declined gradually to 7.0% at month 33 (3.8%, 12.8%). In the control arm, weighted *P. vivax* prevalence declined gradually from 24.1% (15.9%, 34.8%) at baseline to 5.5% (3.2%, 9.2%) at month 33 (Figure 8B). Prevalences in individual clusters reflected the overall trend seen in the arms (Figure 9).

Thus the overall reductions in *P. falciparum* prevalence from baseline to month 33 were 80.7% and 83.7% in the intervention and control villages respectively and the corresponding reductions in *P. vivax* prevalence were 77.1% and 70.0% respectively.

**Figure 10:**
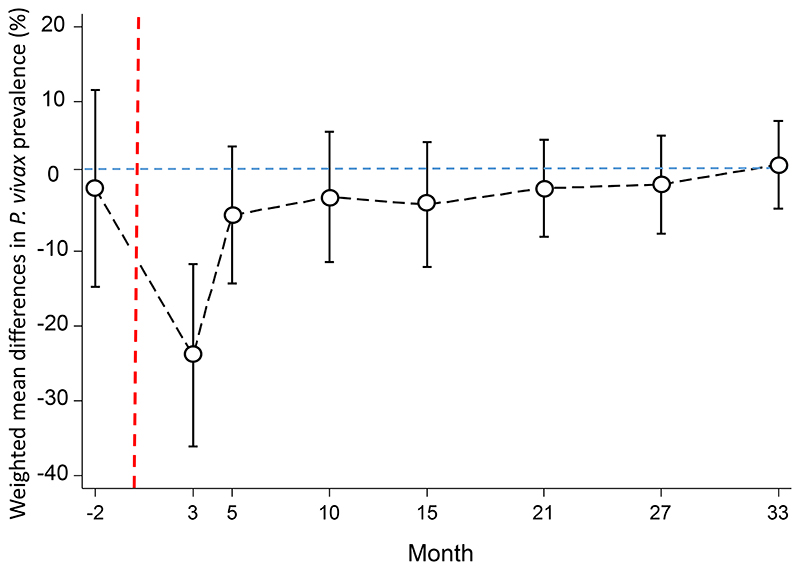
Weighted mean difference in the *P. vivax* prevalence between the MDA relative and control arms. Black circles denote point estimates and capped bars denote the 95% confidence intervals.

Among individuals who received a full course of DHA-piperaquine in month 2, 10/626 (1.6%) had a *P. vivax* infection detected by uPCR at month 3. Immediately after MDA, the intervention arm had a significantly lower prevalence of *P. vivax* infections than the control arm, with a weighted mean difference of -23.9% (95% CI; -36.1, -11.7%, p=0.002, Figure 10). The magnitude of weighted mean difference between the study arms declined at successive time points with a difference of -5.2% (95% CI; -14.3%, 4.0%, p=0.22) at month 5; and +1.6% (95% CI; -4.3, 7.5, p=0.54) at month 33. RDTs identified 0.7% (10/1450) of the uPCR detected *P. vivax* infections.

There were 271 new *P. vivax* infections detected by uPCR in the control arm and 422 new *P. vivax* infections detected in the intervention arm after the MDA. The incidence of newly detected *P. vivax* infections was 257.7 (95% CI: 228.0, 290.3) per 1000 person years in the control arm and 202.9 (184.0, 223.2) in the intervention arm, a weighted mean difference of -79.8 (95% CI: -174.0, 14.4; p=0.0852) new cases.

Weighted *P. malariae* prevalence in the intervention arm was 4.2 (95% CI: 1.8, 9.5) pre-MDA and ≤1% at all timepoints post-MDA (Supplementary Figure 1). Weighted *P. malariae* prevalence remained stable in the control arm at all time points (estimates ranged from 3.0%-4.6%, Supplementary Figure 1). Ten *P. ovale* infections were detected.

### Malaria incidence

**Figure 11:**
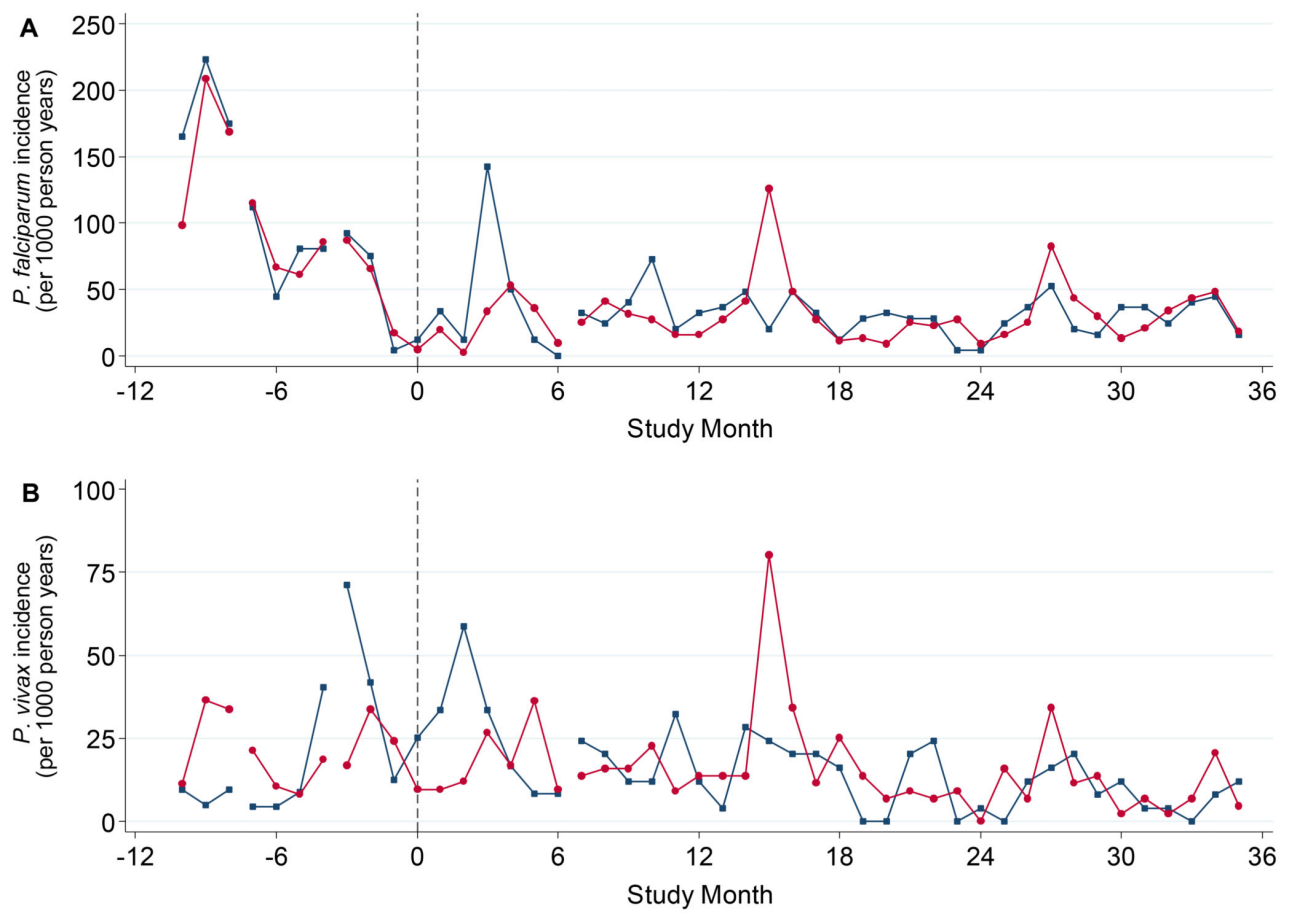
Monthly (A) *P. falciparum* and (B) *P. vivax* incidences in the intervention (red) and control arms (blue). Data were excluded if the matched cluster in the other arm did not yet have an operational CHW. Lines connect time points with the same number of cluster pairs included. 10 clusters provide data from study month -10; 12 from study month -7; 14 from study month-3 and 16 from study month 7. Vertical dashed grey line indicates MDA start (Month 0). For incidence of balanced cohorts of clusters see Supplementary Figures 2-4. For incidence of each individual cluster see Supplementary Figures 5-6.

Fifteen clusters had active trained CHWs in place before the first baseline survey (Month -2). In MDA cluster 1, there were problems recruiting and the CHW started malaria activities only in October 2015 (Month 7). In the year preceding MDA, the incidence of symptomatic *P. falciparum* infections was similar in intervention and control arms (Figure 11A). Among the remaining 14 clusters the *P. falciparum* incidence (cases per 1000 person years) was 90 (95% CI: 81, 99) in the intervention arm and 94 (82, 107) in the control arm. *P. falciparum* incidence was lower in the subsequent years in both MDA and control arms. *P. falciparum* incidence (95% CI) in the MDA arm was 25 (21, 30), 33 (28, 38) and 32 (27, 37) cases per 1000 person years for the first, second and third year post-MDA respectively. Corresponding incidences in the control arm were 38 (31, 46) in year 1, 29 (24, 36) in year 2 and 29 (24, 36) cases per 1000 person years in year 3. In the first year after MDA was concluded, most new symptomatic infections detected by the CHWs in the MDA clusters were among people who did not receive MDA (57%: 31/54).

*P. falciparum* incidence was lower in the MDA intervention arm than in the control arm in the first year after the MDA (weighted mean difference of -11.9 (95% CI: -34.8, 11.0 cases per 1000 person years), though the difference was not statistically significant (p=0.26). In the second and third years post-MDA the difference in *P. falciparum* incidence between study arms had narrowed (weighted mean difference of +1.0 (95% CI: -25.3, 27.3; p=0.93) and -2.3 (95% CI: -35.2, 30.6; p=0.87) cases per 1000 person years respectively). There was considerable heterogeneity in cluster-level falciparum malaria incidence post-MDA; MDA cluster 1 had the highest post-MDA incidence (211 per 1000 person years, while MDA cluster 7 had the lowest post-MDA incidence (0.9 per 1000 person years) (Supplementary Figure 5)

*P. vivax* incidence was lower in the intervention arm than in the control arm in the first year post-MDA (weighted mean difference of -10.2 (95% CI: -35.8, 15.4), though the difference was not statistically significant (p=0.38) (Figure 11B). In the second and third years after the MDA *P. vivax* incidence was similar in both study arms (weighted mean difference of +4.0 (95% CI: -6.9, 14.0; p=0.42) cases and -0.1 (95% CI: -5.3, 5.1; p=0.97) respectively). Overall malaria incidence was lower in the intervention arm than in the control arm in the first year post-MDA (weighted mean difference of -22.1 (95% CI: -56.5, 12.1), though this difference was not statistically significant (p=0.17). In the second and third years post-MDA malaria incidence was similar in both study arms (weighted mean difference of +3.8 (95% CI: -21.0, 28.7; p=0.73) cases and -2.2 (95% CI: -31.5, 27.2; p=0.87) respectively).

### Resistance markers

*Pf* kelch 13 genotyping was successful in 385 of 529 (73%) *P. falciparum* positive blood samples from all survey rounds (Table 2). K13 propeller mutations were identified in 118 of 188 (63%) samples from MDA villages and in 129 of 197 (65%) samples from control villages. In the MDA arm, 28 of 52 (54%) samples had K13 mutations before MDA (month -2) and 7 of 13 (54%) samples had K13 mutations immediately after MDA (month 3). This corresponds to 4.5% of sampled individuals harbouring a K13 mutant infection before MDA and 0.9% immediately after MDA. In the control arm without MDA, the corresponding proportions were 27 of 42 (64%) samples with K13 mutations at month -2 and 31 of 51 (61%) samples at month 3. This corresponds to 6.5% of sampled control individuals harbouring a K13+ infection before and 6.3% five months later. The overall proportion of *P. falciparum* infections with K13 mutations was slightly lower in the MDA arm immediately after MDA (month 3: 54% in MDA arm; 61% in control arm) and at the conclusion of the study (46% in intervention arm; 67% in control arm). F446I was the most prevalent *Pf*-kelch mutant identified comprising 149 of 247 (60%) of K13 mutant infections. Other frequently identified K13 mutations included C580Y (13%), G449A (7%) and N537I (4%). *Plasmepsin 2* copy number genotyping was successful in 299 of 529 (56%) *P. falciparum* positive samples from all survey rounds (Supplementary Table 7). No multi-copy *plasmepsin 2* parasites were observed at any time in the intervention (0/157) or control arms (0/142). Thus there was no evidence that MDA had selected either artemisinin or piperaquine resistant parasites.

### Tolerability and safety

There were 151 adverse events (AEs) reported (Supplementary Table 8-10) during the 3 month MDA period representing 1.4% (151/10,677) of all medication courses distributed and 3.6% (151/4173) of individuals receiving MDA. 120 AEs were classified as mild, 25 as moderate and 6 as severe (Supplementary Table 8). The majority of complaints were dizziness (n=109) and rash or itching (n=20). (Supplementary Table 9). 18 AEs were assessed as probably related to the medication (Supplementary Table 10). Most AEs were in the first treatment round (84) with fewer reported each subsequent round (48 and 19 respectively). Six serious adverse events (SAE) were reported (Supplementary Table 11) including three deaths each of which was assessed as being unlikely to be related to treatment. There were no reports of passing dark urine following primaquine treatment and no sudden unexplained deaths in otherwise healthy individuals which might have resulted from cardiac arrhythmias.

## Discussion

In the Greater Mekong sub-region (GMS), as in much of the malaria endemic world, malaria transmission is low and heterogeneous. In the GMS the main anopheline vectors often bite outdoors at dusk or dawn and conventional vector control measures (insecticide treated bed nets, insecticide spraying) are less effective than elsewhere. This places a greater reliance on antimalarial drugs for malaria control. In recent years in the eastern GMS, and along the Thailand-Myanmar border, the efficacy of the first line ACTs has declined alarmingly as first artemisinin resistance, and then partner drug resistance, has increased and spread ^6, 9, 10, 39, 40^. There is therefore a race to eliminate malaria in the GMS before resistance prevents it. Fortunately in most of Myanmar, which bears the main burden of malaria in the region, ACT partner drug (lumefantrine) resistance has not yet emerged ^11^. Malaria in forest and forest fringe communities is unevenly distributed with some villages having a high prevalence of asymptomatic infections. This study shows that implementation and support of community health workers (CHW) to diagnose and treat malaria in these remote malaria affected villages followed by antimalarial mass drug administration (MDA) with dihydroartemisinin-piperaquine can safely and rapidly reduce the burden of *P. falciparum* despite a high prevalence of artemisinin resistance. MDA reduced asymptomatic *P. falciparum* prevalence by 90% in the first year, then early diagnosis and treatment of symptomatic malaria cases by the CHWs plus vector control maintained low levels of *P. falciparum* infection for nearly three years.

This combination of malaria control activities is necessary for sustained success. It requires uninterrupted support for the CHWs to provide early diagnosis and appropriate antimalarial treatment (EDT) and a strong community engagement programme ^33^ in order to achieve a high population coverage and concomitant vector control. The properly supported CHW is the key to malaria control in this region. In this long prospective study conducted in remote areas of South-Eastern Myanmar well-supported and well-functioning CHWs reduced malaria by a factor of four over a three year period. These reductions in malaria prevalence are faster than reported previously with implementation of community based EDT and vector control programmes ^41–43^.The rapidity of the malaria decline in our study may have been increased by the intermittent RDT screening and treatment provided (i.e. active case detection of people with higher parasitaemias) at each survey time point, although this only accounted for a total of 37 treatments given across eight surveys. Spillover benefits from ACTs delivered to the neighbouring MDA villages could also be a contributory factor. The paired study design meant that the intervention and control clusters were in close proximity and so control clusters would have experienced less importation of *P. falciparum* parasites from neighbouring MDA clusters than before. However the most isolated control village, far from any MDA village also experienced a protracted reduction in malaria prevalence indicating that community based EDT and LLIN interventions reduce the asymptomatic reservoir as well. Notably the cluster with the poorest results (i.e. highest *P. falciparum* incidence after MDA) was without an active CHW until 7 months after MDA had begun.

MDA is a malaria elimination accelerator. Two factors determine MDA efficacy – coverage and efficacy. In this study 10% of the population did not receive MDA (either because they refused, or because they were not eligible to receive it) providing an important residual reservoir of infection. Driving low levels of falciparum malaria to elimination requires prevention of reintroduction. Even in these remote forested areas people movement was extensive. MDA clusters with the lowest *P. falciparum* prevalence after MDA were the more isolated clusters. Isolation presumably guarded them from reintroduction of *P. falciparum* from neighbouring untreated communities. Reintroduction confounds small studies but in a programmatic implementation of MDA, ideally all malaria hotspots in a target area would be treated. This would reduce reintroduction form adjacent areas and so the benefit conferred by MDA should be greater than shown here. Systematic large scale MDA distribution is feasible. In part of Eastern Myanmar where a similar approach has been prosecuted there has been no significant rebound of *P. falciparum* prevalence for 12 months after implementing MDA ^44, 45^.

We delivered >10,000 courses of DP to healthy subjects using DOT, of which >99% were completed. MDA with this ACT proved well tolerated safe and was widely accepted in this evaluation. This confirms previous reports that large scale administration of multiple courses of DP is acceptable and safe ^46^.

The high efficacy of dihydroartemisinin-piperaquine in artemisinin resistant infections is unsurprising as piperaquine remains highly effective in Myanmar. There was no evidence for the plasmepsin amplification associated with piperaquine resistance in the Eastern GMS ^34^. MDA usually encounters relatively low parasite densities which have already been controlled by immunity, which together improve the chance of successful parasite clearance regardless of resistance. Piperaquine has a long terminal elimination half-life and monthly repeated courses ensure that MDA participants have only a single time period, after the final treatment round, where newly introduced malaria parasites may be exposed to sub-therapeutic drug concentrations. By that time most *P.falciparum* parasites have disappeared from the community because of MDA. Unsurprisingly there was no evidence for the selection of drug resistant parasites in this study.

Four people (0.6%) had *P. falciparum* and ten (1.6%) had *P. vivax* detected in blood samples one month after receiving a full treatment course of dihydroartemisinin-piperaquine. Without drug level measurement it is not possible to determine whether or not these were treatment failures. uPCR detects reliably parasite densities as low as 22/mL, which means that the parasitaemia in the first asexual cycle following liver schizont rupture could be detected. As the MDA drugs used do not affect pre-erythrocytic development, and are not hypnozoitocidal, it is possible that some of the parasitaemias detected one month after MDA resulted from recent pre-erythrocytic schizogony.

In contrast to *P. falciparum*, and as observed elsewhere, the initial substantial reduction of *P. vivax* prevalence at month 3 was not maintained. This was anticipated as *P. vivax* hypnozoites were not treated by the MDA regimen of DP and a single dose of PQ and relapses of vivax malaria were expected. On the other hand, the *P. malariae* prevalence reduction after MDA was sustained reflecting the absence of hypnozoites in this species. The gradual decline in the prevalence of uPCR detectable *P. vivax* in the control arm mirrors results reported recently from an observational study in Myanmar that described substantial declines in clinical *P. vivax* incidence and positivity rates in communities receiving community based case management by CHWs ^14^.

A limitation of this study is the small number of randomised clusters which meant that an assessment of the impact of cluster-level covariates was not feasible. By its nature, mass drug administration cannot be studied in an individual randomised control trial. The paired design in this trial, where control hotspots were left untreated, limits the generalizability of this study as programmatic implementation of MDA would likely take place across a contiguous and large region. Prevalence surveys were conducted in adults only as we were not permitted to take blood repeatedly from children.

Historically, MDA has often led to an initial reduction in malaria outcomes, but reductions are often not sustained, or there has been no long-term follow up ^22^. MDA regimens have been successful in eliminating malaria in island settings in Vanuatu and the Comoros ^23, 47^ and recent studies from elsewhere in the GMS have provided promising results ^44, 48^. Effective MDA requires a significant political, logistical and financial commitment. It should be considered an accelerator of elimination, and not a substitute for community based EDT. The poor long-term outcome in the MDA cluster with no active CHW in the seven months post-MDA highlights the limits of conducting MDA without the inclusion of routine control measures. While the drugs remain effective MDA is a safe and effective tool to accelerate *P. falciparum* elimination, alongside routine malaria control measures.

## Supplementary Material

Supplementary Materials

## Figures and Tables

**Table 1:** Baseline characteristics of MDA intervention and corresponding control clusters

			Intervention Clusters									Control Clusters		
	Census N	Female	Age (years)	uPCR N	*P. falciparum* infections	*P. vivax* infections	Un-speciated malaria	Total malaria infections	Census N	Female	Age (years)	uPCR N	*P. falciparum* infections	*P. vivax* infections
**Pair 1**	283	136 (48)	16 [7,31]	57	15 (26)	21 (37)	4 (7)	39 (68)	132	73 (55)	20 [9,41]	33	10 (30)	7 (21)
**Pair 2**	468	244 (52)	14 [7,25]	63	22 (35)	23 (37)	0 (0)	39 (62)	189	102 (54)	19 [9,37]	37	10 (27)	6 (16)
**Pair 3**	246	125 (51)	19 [9,36]	55	7 (13)	19 (35)	0 (0)	24 (44)	208	103 (50)	17 [8,33]	49	9 (18)	14 (29)
**Pair 4**	313	152 (49)	18 [7,34]	51	10 (20)	8 (16)	0 (0)	20 (39)	320	171 (53)	16.5 [6,32]	59	11 (19)	23 (39)
**Pair 5**	1279	609 (48)	22 [9,40]	184	23 (13)	41 (22)	5 (3)	74 (40)	545	276 (51)	19 [7,35]	66	13 (20)	22 (33)
**Pair 6**	810	414 (51)	18 [9,35]	76	11 (15)	20 (27)	2 (3)	38 (50)	816	412 (50)	20 [10,38]	72	12 (17)	9 (13)
**Pair 7**	1135	650 (57)	19 [8,36]	76	8 (11)	5 (7)	1 (1)	13 (17)	707	349 (49)	22 [10,38]	60	5 (8)	11 (18)
**Pair 8**	947	475 (50)	19 [9,38]	75	5 (7)	13 (17)	4 (5)	25 (33)	323	150 (46)	18 [7,35]	55	4 (7)	14 (25)
**Total**	5481	2805 (51)	19 [8,36]	637	101 (16)	150 (24)	16 (3)	272 (43)	3240	1636 (50)	20 [8,36]	431	74 (17)	106 (25)

n (%) and medians [25^th^, 75^th^ percentile] are presented. uPCR = ultrasensitive high volume Polymerase Chain Reaction quantitative malaria test. Percentages in total rows are unweighted.

**Table 2:** Kelch13 genotypes of *P. falciparum* positive samples from all survey rounds

	Screening (Month -2)		Month 3		Month 5		Month 10		Month 15		Month 21		Month 27		Month 33	
	MDA	Control	MDA	Control	MDA	Control	MDA	Control	MDA	Control	MDA	Control	MDA	Control	MDA	Control
**N (Successful Amplification)**	52	42	13	51	14	17	20	24	32	21	11	14	20	13	26	15
K13 Wild Type	24 (46)	15 (36)	6 (46)	20 (39)	3 (21)	5 (29)	7 (35)	11 (46)	6 (19)	6 (29)	4 (36)	4 (29)	6 (30)	2 (15)	14 (54)	5 (33)
K13 mutation	28 (54)	27 (64)	7 (54)	31 (61)	11 (79)	12 (71)	13 (65)	13 (54)	26 (81)	15 (71)	7 (64)	10 (71)	14 (70)	11 (85)	12 (46)	10 (67)
F446I^a^	10 (19)	12 (29)	4 (31)	18 (35)	9 (64)	8 (47)	5 (25)	4 (17)	17 (53)	10 (48)	4 (36)	8 (57)	13 (65)	9 (69)	11 (42)	7 (47)
C580Y^a^	5 (10)	4 (10)	2 (15)	4 (8)	1 (7)	2 (12)	2 (10)	3 (13)	1 (3)	3 (14)	1 (9)		1 (5)	1 (8)	1 (4)	2 (13)
G449A^b^	4 (8)			2 (4)			4 (20)	1 (4)	4 (13)	1 (5)						1 (7)
N537I^b^	1 (2)	5 (12)		2 (4)		1 (6)						1 (7)				
C469F^b^	1 (2)	1 (2)		1 (2)			2 (10)	2 (8)	1 (3)		1 (9)					
P553L^a^	3 (6)	2 (5)							1 (3)		1 (9)					
K438N	2 (4)	2 (5)		1 (2)		1 (6)		1 (4)								
P574L^b^			1 (8)	1 (2)	1 (11)			1 (4)				1 (7)		1 (8)		
R561H^a^		1 (2)						1 (4)		1 (5)						
I205T	1 (2)															
R575K^c^	1 (2)															
K586E/K				1 (2)												
M476I^a^				1 (2)												
E321K									1 (3)							
R528S/R									1 (3)							

n (%) presented. K13 mutations are arranged in order of frequency of detection.

a Validated resistance mutation ^38^

b Candidate resistance mutation ^38^

c Reported to be associated with delayed clearance but not statistically significant ^38^
