# Supplementary material for: Mass drug administration for the acceleration of malaria elimination in a region of Myanmar with artemisinin-resistant falciparum malaria: a cluster-randomised trial
Source: Lancet Infect Dis. Author manuscript; Available in PMC 2023 May 5. (PMC7614510; doi:10.1016/S1473-3099(20)30997-X)
Supplement: Supplementary Materials [file EMS171004-supplement-Supplementary_Materials.pdf]

Supplementary Materials

Supplementary table 1. Results from baseline uPCR survey in 43 villages screened.

Samples	*P. falciparum*	*P. vivax*	*P. ovale*	*P. malariae*	Plasmodium spp.
67	10 (15)	16 (24)	0 (0)	0 (0)	2 (3)
56	11 (20)	9 (16)	0 (0)	2 (4)	0 (0)
44	1 (2)	3 (7)	0 (0)	0 (0)	0 (0)
72	12 (17)	9 (13)	1 (1)	1 (1)	8 (11)
76	8 (11)	5 (7)	0 (0)	1 (1)	1 (1)
33	10 (30)	7 (21)	0 (0)	4 (12)	2 (6)
59	16 (27)	22 (37)	0 (0)	10 (17)	4 (7)
39	1 (3)	5 (13)	1 (3)	0 (0)	2 (5)
66	13 (20)	25 (38)	0 (0)	4 (6)	5 (8)
59	11 (19)	23 (39)	0 (0)	8 (14)	1 (2)
65	8 (12)	19 (29)	0 (0)	0 (0)	2 (3)
69	1 (1)	1 (1)	0 (0)	0 (0)	1 (1)
68	0 (0)	5 (7)	0 (0)	0 (0)	0 (0)
76	11 (14)	22 (29)	0 (0)	7 (9)	2 (3)
78	6 (8)	15 (19)	0 (0)	3 (4)	4 (5)
54	5 (9)	16 (30)	0 (0)	1 (2)	2 (4)
67	1 (1)	10 (15)	0 (0)	0 (0)	1 (1)
64	3 (5)	10 (16)	0 (0)	0 (0)	0 (0)
68	3 (4)	17 (25)	0 (0)	2 (3)	0 (0)
49	9 (18)	14 (29)	0 (0)	2 (4)	2 (4)
64	1 (2)	12 (19)	0 (0)	0 (0)	0 (0)
55	7 (13)	19 (35)	0 (0)	0 (0)	0 (0)
37	10 (27)	6 (16)	1 (3)	0 (0)	1 (3)
55	0 (0)	15 (27)	0 (0)	0 (0)	2 (4)
47	2 (4)	19 (40)	0 (0)	1 (2)	0 (0)
63	22 (35)	24 (38)	0 (0)	7 (11)	0 (0)
65	0 (0)	7 (11)	0 (0)	0 (0)	0 (0)
84	0 (0)	9 (11)	0 (0)	0 (0)	3 (4)
62	26 (42)	6 (10)	0 (0)	4 (6)	11 (18)
76	2 (3)	10 (13)	0 (0)	1 (1)	1 (1)
56	0 (0)	9 (16)	0 (0)	0 (0)	2 (4)
60	5 (8)	11 (18)	0 (0)	2 (3)	0 (0)
59	0 (0)	1 (2)	0 (0)	0 (0)	0 (0)
75	0 (0)	4 (5)	0 (0)	0 (0)	0 (0)
77	1 (1)	12 (16)	0 (0)	2 (3)	5 (6)
64	0 (0)	6 (9)	0 (0)	1 (2)	0 (0)
67	3 (4)	21 (31)	0 (0)	7 (10)	3 (4)
75	1 (1)	5 (7)	0 (0)	0 (0)	5 (7)
55	4 (7)	14 (25)	0 (0)	3 (5)	0 (0)
66	1 (2)	9 (14)	0 (0)	0 (0)	2 (3)
57	5 (9)	13 (23)	0 (0)	1 (2)	0 (0)
68	2 (3)	13 (19)	0 (0)	0 (0)	2 (3)
58	2 (3)	5 (9)	0 (0)	0 (0)	0 (0)
2674	234 (9)	503 (19)	3 (0)	74 (3)	76 (3)

n (%) are presented.

Supplementary Table 2. Overall coverage of MDA in intervention clusters, n (% of present) population)

MDA Cluster	Present[a]	Eligible	At least 1 course	Received 3 courses	Received 2 courses only[b]	Single course only[c]	Partial course only	Refused	<1y old	Pregnant 1st trimester	Ine ot
1	215	204 (94.9)	196 (91.2)	117 (54.4)	27 (12.6)	52 (24.2)	2 (0.9)	6 (2.8)	8 (3.7)	2 (0.9)	1
2	450	432 (96)	337 (74.9)	189 (42)	70 (15.6)	78 (17.3)	1 (0.2)	94 (20.9)	17 (3.8)	0 (0)	1
3	222	216 (97.3)	203 (91.4)	173 (77.9)	19 (8.6)	11 (5)	0 (0)	13 (5.9)	5 (2.3)	0 (0)	1
4	266	256 (96.2)	251 (94.4)	188 (70.7)	35 (13.2)	28 (10.5)	0 (0)	5 (1.9)	8 (3)	1 (0.4)	1
5	1045	1014 (97)	985 (94.3)	699 (66.9)	166 (15.9)	120 (11.5)	1 (0.1)	28 (2.7)	30 (2.9)	1 (0.1)	0
6	574	548 (95.5)	515 (89.7)	308 (53.7)	114 (19.9)	93 (16.2)	5 (0.9)	28 (4.9)	9 (1.6)	4 (0.7)	13
7	1052	1013 (96.3)	955 (90.8)	731 (69.5)	132 (12.5)	92 (8.7)	3 (0.3)	55 (5.2)	38 (3.6)	1 (0.1)	0
8	794	782 (98.5)	714 (89.9)	492 (62)	154 (19.4)	68 (8.6)	5 (0.6)	63 (7.9)	9 (1.1)	2 (0.3)	1
All MDA	4618	4465 (96.7)	4156 (90.0)	2897 (62.7)	717 (15.5)	542 (11.7)	17 (0.4)	292 (6.3)	124 (2.7)	11 (0.2)	18

[a] Physically present in the target cluster on at least one day of MDA distribution

[b] Completed at least 2 but not 3 MDA treatment courses

[c] Completed at least 1 but not 2 or more MDA treatment courses

[d] Including previous adverse reaction to medication and concurrent illness

Supplementary Table 3. MDA coverage by round and cluster

	MDA Cluster	Present in Round	Eligible for MDA	Completed MDA	Refused all medication in round	Ineligible for medication	Under 1 year old	Pregnant 1st trimester	Other ineligible	Partial course in round	Took 1 or 2 doses then refused	Took 1 or 2 doses then deemed ineligible	To[illegible] then[illegible] fo[illegible]
M0	1	180	166 (92.2)	149 (82.8)	13 (7.2)	14 (7.8)	9 (5)	4 (2.2)	1 (0.6)	4 (2.2)	3 (1.7)	1 (0.6)	
	2	380	358 (94.2)	255 (67.1)	102 (26.8)	22 (5.8)	17 (4.5)	2 (0.5)	3 (0.8)	1 (0.3)	1 (0.3)	0 (0)	
	3	197	190 (96.4)	174 (88.3)	16 (8.1)	7 (3.6)	5 (2.5)	2 (1)	0 (0)	0 (0)	0 (0)	0 (0)	
	4	230	220 (95.7)	214 (93)	6 (2.6)	10 (4.3)	8 (3.5)	1 (0.4)	1 (0.4)	0 (0)	0 (0)	0 (0)	
	5	844	807 (95.6)	764 (90.5)	39 (4.6)	37 (4.4)	30 (3.6)	6 (0.7)	1 (0.1)	4 (0.5)	2 (0.2)	0 (0)	
	6	475	442 (93.1)	402 (84.6)	33 (6.9)	34 (7.2)	12 (2.5)	5 (1.1)	16 (3.4)	6 (1.3)	1 (0.2)	1 (0.2)	
	7	966	923 (95.5)	834 (86.3)	84 (8.7)	42 (4.3)	39 (4)	3 (0.3)	1 (0.1)	6 (0.6)	3 (0.3)	0 (0)	
	8	728	715 (98.2)	640 (87.9)	68 (9.3)	13 (1.8)	9 (1.2)	2 (0.3)	2 (0.3)	7 (1)	3 (0.4)	0 (0)	
	Total M0	4000	3821 (95.5)	3432 (85.8)	361 (9)	179 (4.5)	129 (3.2)	25 (0.6)	25 (0.6)	28 (0.7)	13 (0.3)	2 (0.1)	1[illegible]
M1	1	176	163 (92.6)	142 (80.7)	20 (11.4)	13 (7.4)	9 (5.1)	4 (2.3)	0 (0)	1 (0.6)	0 (0)	0 (0)	
	2	376	356 (94.7)	257 (68.4)	99 (26.3)	20 (5.3)	17 (4.5)	2 (0.5)	1 (0.3)	0 (0)	0 (0)	0 (0)	
	3	213	208 (97.7)	194 (91.1)	14 (6.6)	5 (2.3)	5 (2.3)	0 (0)	0 (0)	0 (0)	0 (0)	0 (0)	
	4	233	222 (95.3)	215 (92.3)	7 (3)	11 (4.7)	8 (3.4)	2 (0.9)	1 (0.4)	0 (0)	0 (0)	0 (0)	
	5	935	902 (96.5)	864 (92.4)	38 (4.1)	33 (3.5)	30 (3.2)	3 (0.3)	0 (0)	0 (0)	0 (0)	0 (0)	
	6	498	475 (95.4)	421 (84.5)	47 (9.4)	23 (4.6)	9 (1.8)	5 (1)	9 (1.8)	7 (1.4)	0 (0)	3 (0.6)	
	7	982	937 (95.4)	856 (87.2)	74 (7.5)	45 (4.6)	39 (4)	6 (0.6)	0 (0)	7 (0.7)	0 (0)	0 (0)	
	8	691	679 (98.3)	593 (85.8)	82 (11.9)	12 (1.7)	9 (1.3)	2 (0.3)	1 (0.1)	4 (0.6)	0 (0)	0 (0)	
	Total M1	4104	3942 (96.1)	3542 (86.3)	381 (9.3)	162 (3.9)	126 (3.1)	24 (0.6)	12 (0.3)	19 (0.5)	0 (0)	3 (0.1)	1[illegible]
M2	1	192	181 (94.3)	166 (86.5)	14 (7.3)	11 (5.7)	8 (4.2)	3 (1.6)	0 (0)	1 (0.5)	0 (0)	0 (0)	
	2	392	374 (95.4)	273 (69.6)	100 (25.5)	18 (4.6)	17 (4.3)	1 (0.3)	0 (0)	1 (0.3)	0 (0)	0 (0)	

3	213	208 (97.7)	200 (93.9)	8 (3.8)	5 (2.3)	5 (2.3)	0 (0)	0 (0)	0 (0)	0 (0)	0 (0)
4	240	227 (94.6)	219 (91.3)	7 (2.9)	13 (5.4)	8 (3.3)	3 (1.3)	2 (0.8)	1 (0.4)	1 (0.4)	0 (0)
5	982	950 (96.7)	921 (93.8)	29 (3)	32 (3.3)	30 (3.1)	2 (0.2)	0 (0)	0 (0)	0 (0)	0 (0)
6	498	476 (95.6)	422 (84.7)	53 (10.6)	22 (4.4)	9 (1.8)	5 (1)	8 (1.6)	1 (0.2)	0 (0)	0 (0)
7	972	928 (95.5)	859 (88.4)	67 (6.9)	44 (4.5)	38 (3.9)	6 (0.6)	0 (0)	2 (0.2)	0 (0)	0 (0)
8	687	672 (97.8)	587 (85.4)	82 (11.9)	15 (2.2)	9 (1.3)	2 (0.3)	4 (0.6)	3 (0.4)	0 (0)	0 (0)
Total M2	4176	4016 (96.2)	3647 (87.3)	360 (8.6)	160 (3.8)	124 (3)	22 (0.5)	14 (0.3)	9 (0.2)	1 (0)	0 (0)

Supplementary Table 4. Odds ratios (95% Confidence Intervals) from exploratory analyses of factors associated with uPCR detected *P. falciparum* infection in intervention clusters after MDA.

Variable	Month 3	Month 5	Month 10	Month 15	Month 21	Month 27	Month 33
Male	5.5 (1.2,25.6)	3.2 (0.9,11.8)	2.0 (1.0,4.2)	3.9 (1.7,9.3)	4.1 (1.1,15.1)	1.6 (0.8,3.4)	2.8 (1.2,6.5)
Age (per 10 years)	1.3 (0.7,2.1)	0.9 (0.6,1.5)	0.9 (0.7,1.2)	1.0 (0.8,1.4)	0.9 (0.6,1.4)	0.8 (0.6,1.1)	1.0 (0.8,1.4)
Reported recent[a] overnight forest stay?	3.2 (0.9,11.2)	2.8 (0.9,8.7)	2.1 (1.0,4.3)	1.9 (0.9, 3.9)	1.8 (0.6,5.4)	2.2 (1.0,4.9)	2.1 (1.0,4.4)
Reported recent[a] overnight stay outside[b] of the village?	0.5 (0.1,2.4)	2.3 (0.7,7.3)	1.6 (0.8,3.4)	1.2 (0.6,2.7)	0.2 (0.03,1.9)	1.0 (0.4,2.3)	1.2 (0.5,2.7)
Reported frequency of bednet use							
Never	Reference	Reference	Reference	Reference	No events	Reference	Reference
Sometimes [c]	1.8 (0.2,16.6)	0.3 (0.04,1.8)	0.8 (0.2,3.0)	1.1 (0.3,3.9)	Reference	0.9 (0.1,5.9)	0.5 (0.2,1.9)
Usually [d]	1.3 (0.1, 11.2)	0.2 (0.05,0.9)	0.4 (0.1,1.1)	0.8 (0.2,2.7)	0.2 (0.1,0.5)	0.9 (0.2,4.2)	0.3 (0.1,0.9)
Did not receive MDA?	19.0 (5.6,64.7)	5.0 (1.6,16.0)	2.1 (1.0,4.5)	1.4 (0.7,3.0)	3.3 (1.1,9.7)	2.0 (0.9,4.3)	2.6 (1.3,5.4)

Odds Ratios (95% Confidence Intervals) are presented from univariable multilevel mixed-effects model with cluster as a random effect.

[a] Question wording changed from survey to survey so the definition of “recent” varies across surveys (see Supplementary Table 5).

[b] Question wording changed from survey to survey so the definition of “outside” varies across (see Supplementary Table 5).

[c] 1-3 nights per week. Question wording and order changed from survey to survey (see Supplementary Table 6).

[d] Question wording and order changed from survey to survey so the definition of “usually” varies across surveys from (see Supplementary Table 6).

Supplementary Table 5. Wording of questions for uPCR surveys included as an exposure in the model reported in Table 1 and Table 2

Month	Reported recent overnight forest stay	Reported recent overnight stay outside of the village
-2	Did you stay overnight in the forest in last 3 months?	Did you travel outside of village overnight in the last 3 months?
3	Did you stay overnight in the forest during last 5 months (since we took blood from you)?	Did you travel and stay overnight outside of village (but not the same forest under Q4) during the last 5 months?
5	Did you stay overnight in the forest since January (or June) 2015 (since we took blood from you)?	Did you travel and stay overnight outside of village (but not the same forest under Q4) since January (or June) 2015 ?
10	Did you stay overnight in the forest since May (beginning of rainy season) 2015?	Did you travel and stay overnight outside of village (but not the same forest under Q4) since May (Beginning of rainy season) 2015?
15	Did you stay overnight in the forest since May (beginning of rainy season) 2015, or your previous uPCR test?	Did you travel and stay overnight outside of village (but not the same forest under Q5) since May 2015 or your previous uPCR test?
21	Did you stay overnight in the forest in the last 3 months?	Did you stay overnight in another village/town in last 3 months?
27	Did you stay overnight in the forest in the last 3 months?	Did you stay overnight in another village/town in last 3 months?
33	Did you stay overnight in the forest in the last 3 months?	Did you stay overnight in another village/town in last 3 months?

Supplementary Table 6. Wording of questions for bednet use frequency

Month	Frequency of use	Ownership	Ownership asked before or after frequency asked
-2	**Do you use a bed net?**<br>Most nights [a]<br>Some nights [b]<br>Never use [c]		
3	**Do you use a bed net?**<br>Yes, use 4-7 nights per week [a]<br>Yes, use 1-3 nights per week [b]<br>No, never use [c]	**If not using a bed net: do you /your family have a bed net?**<br>Yes<br>No [c]	After
5	**How often do you use bed nets?**<br>Yes, use every night [a]<br>Yes use 4-6 nights per week [a]<br>Yes, use 1-3 nights per week [b]<br>No, never use [c]	**Do you have a bed net?**<br>Yes<br>No [c]	Before
10	**How often do you use bed nets?**<br>Yes, use every night [a]<br>Yes use 4-6 nights per week [a]<br>Yes, use 1-3 nights per week [b]<br>No, never use [c]	**Do you have a bed net?**<br>Yes<br>No [c]	Before
15	**How often do you use bed nets?**<br>Yes, use every night [a]<br>Yes use 4-6 nights per week [a]<br>Yes, use 1-3 nights per week [b]<br>No, never use [c]	**Do you have a bed net?**<br>Yes<br>No [c]	Before
21	**Do you use a bed net?**<br>Yes, everyday [a]<br>Yes, 4-6 nights per week [a]<br>Yes, use 1-3 nights per week [b]<br>No, never use [c]		
27	**Do you use a bed net?**<br>Yes, everyday [a]<br>Yes, 4-6 nights per week [a]<br>Yes, use 1-3 nights per week [b]<br>No, never use [c]		
33	**Do you use a bed net?**<br>Yes, everyday [a]<br>Yes, 4-6 nights per week [a]<br>Yes, use 1-3 nights per week [b]<br>No, never use [c]		

[a] Response coded to usually use

[b] Response coded to sometimes use

[c] Response coded to never use

Supplementary Table 7. *Plasmepsin 2* copy number

	**Screening (Month -2)**		**Month 3**		**Month 5**		**Month 10**		**Month 15**		**Month 21**		**Month 27**		**Month 33**	
	MDA	Control	MDA	Control	MDA	Control	MDA	Control	MDA	Control	MDA	Control	MDA	Control	MDA	Control
**Multicopy plasmepsin 2**	0/50	0/41	0/7	0/27	0/6	0/10	0/18	0/14	0/26	0/14	0/7	0/9	0/19	0/14	0/24	0/13
**Median Copy Number Variation (relative quantity)**	0.98	0.92	0.92	0.96	0.97	0.98	0.98	0.99	0.96	0.88	0.93	0.96	1.09	1.11	1.00	1.02

Supplementary table 8. Adverse events by MDA round and severity

Severity	Overall, n (%)	Round 1, n (%)	Round 2, n (%)	Round 3, n (%)
Mild	120 (79.5)	66 (78.6)	42 (87.5)	12 (63.2)
Moderate	25 (16.6)	17 (20.2)	5 (10.4)	3 (15.8)
Severe	6 (4)	1 (1.2)	1 (2.1)	4 (21.1)
Total	151	84	48	19

Supplementary table 9. Classification of adverse events

Types	Round 1	Round 2	Round 3	Total
Dizziness	62 (73.8)	40 (83.3)	7 (36.8)	109 (72.2)
Rash and itchiness	11 (13.1)	5 (10.4)	4 (21.1)	20 (13.2)
Nausea	2 (2.4)			2 (1.3)
Vomiting	7 (8.3)		1 (5.3)	8 (5.3)
Abdominal pain	1 (1.2)		1 (5.3)	2 (1.3)
Others	1 (1.2)	3 (6.3)	6 (31.6)	10 (6.6)
Bleeding per vagina			2 (10.5)	2 (1.3)
Fever	1 (1.2)	1 (2.1)	4 (21.1)	6 (4)
Headache		1 (2.1)		1 (0.7)
Hypertension		1 (2.1)		1 (0.7)
Total	84 (100)	48 (100)	19 (100)	151 (100)

Supplementary table 10. Adverse events categorised by relation to medication

Relationship to drug	n (%)
Not	12 (7.9)
Unlikely	40 (26.5)
Possibly	81 (53.6)
Probably	18 (11.9)
Total	151

Supplementary Table 11. Serious Adverse Events

SAE number	Age (years)	Gender	N rounds MDA completed	Date last took DP (±PQ)	Date of Onset	Description	Outcome	Trial drug relationship	Comments
SAE001	3	F	1	6-Mar-2015	6-Mar-2015	Severe dehydration secondary to diarrhoea and vomiting which started 4 h after taking study drugs. Judged to require hospitalization but parents refused	Recovered	Unknown	Continued study drugs without problem
SAE002	56	M	2	5-Apr-2015	6-Apr-2015	Sudden death (collapse) while exerting himself (cutting down a tree)	Death	Unlikely	Known hypertension- on enalapril for 3 years
SAE003	74	F	2	1-Apr-2015	10-Apr-2015	Death on 24-Apr-2015 following two weeks of abdominal pain similar to pre-existing symptoms and anorexia	Death	Unlikely	Weight 37.2 kg. Long history of gastritis
SAE004	19	F	2	1-Apr-2015	24-Apr-2015	Stillbirth on 29 April 2015 after 5 days vaginal bleeding (self-reported gestation = 7 months)	Recovered	Unlikely	Gravida 1, Parity 0. DP without PQ given<br>Did not receive 3rd round
SAE005	33	F	3	4-May-2015	5-May 2015	Shock secondary to incomplete abortion requiring hospitalization for evacuation of retained products of conception and blood transfusion	Recovered	Possible	G9, P6. Did not know she was pregnant.
SAE006	12	F	2	2-Apr-2015	8-Apr-2015	Death on 9-Apr-2018 after short (<24h) illness: small haematemesis and generalised itching (no rash or jaundice).	Death	Unlikely	

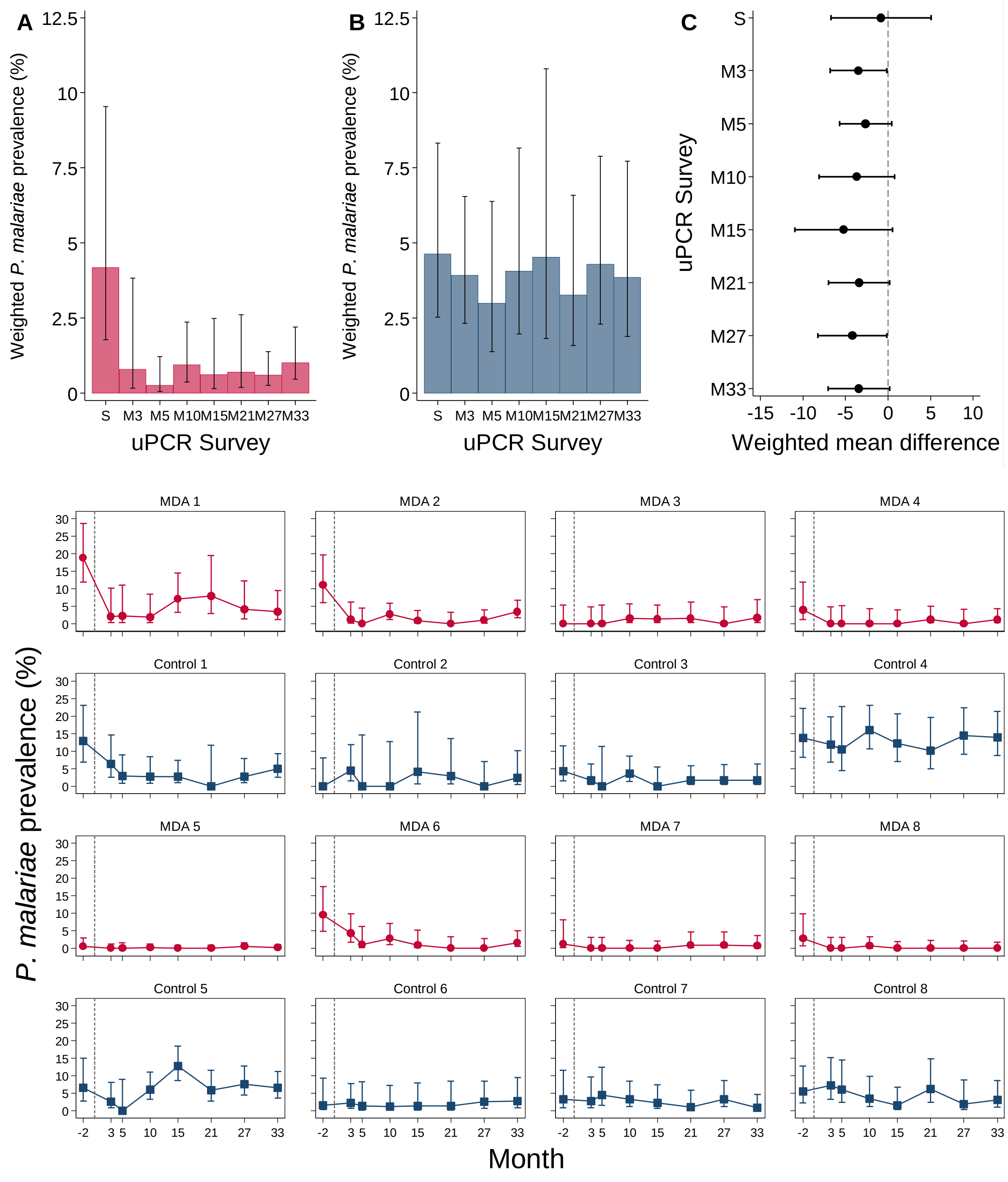

Supplementary Figure 1. *P. malariae* prevalence (by uPCR). Weighted mean *P. malariae* prevalence by uPCR survey timepoint in (A) intervention arm and (B) control arm. Red (intervention) and blue (control) bars denote point estimate and vertical capped lines indicate 95% confidence intervals. (C) Weighted mean difference (black dots) and 95% confidence intervals (horizontal capped lines) in *P. malariae* prevalence in intervention arm relative to control arm by uPCR survey timepoint. Dashed grey line denotes weighted mean difference of zero. (D) *P. malariae* prevalence in intervention and control clusters over time. Red circles (intervention) and blue squares (control) denote point estimates and capped vertical lines denote 95% confidence intervals. Vertical dashed grey line indicates MDA start (Month 0). S = screening survey; M = survey month.

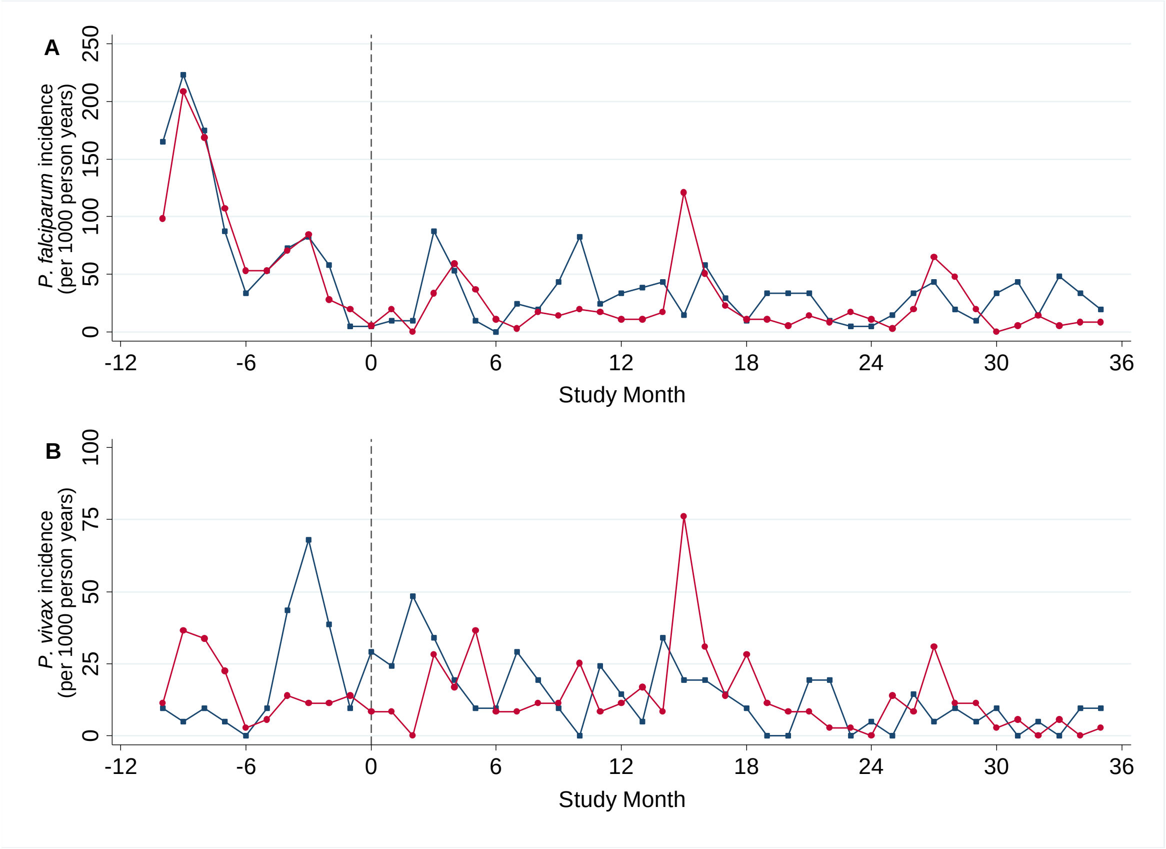

Supplementary Figure 2. Monthly (A) *P. falciparum* and (B) *P. vivax* incidence in intervention (red) and control arm (blue) in study pairs 4-8. Study pairs 4 through 8 all had operational CHWs from study month -10.

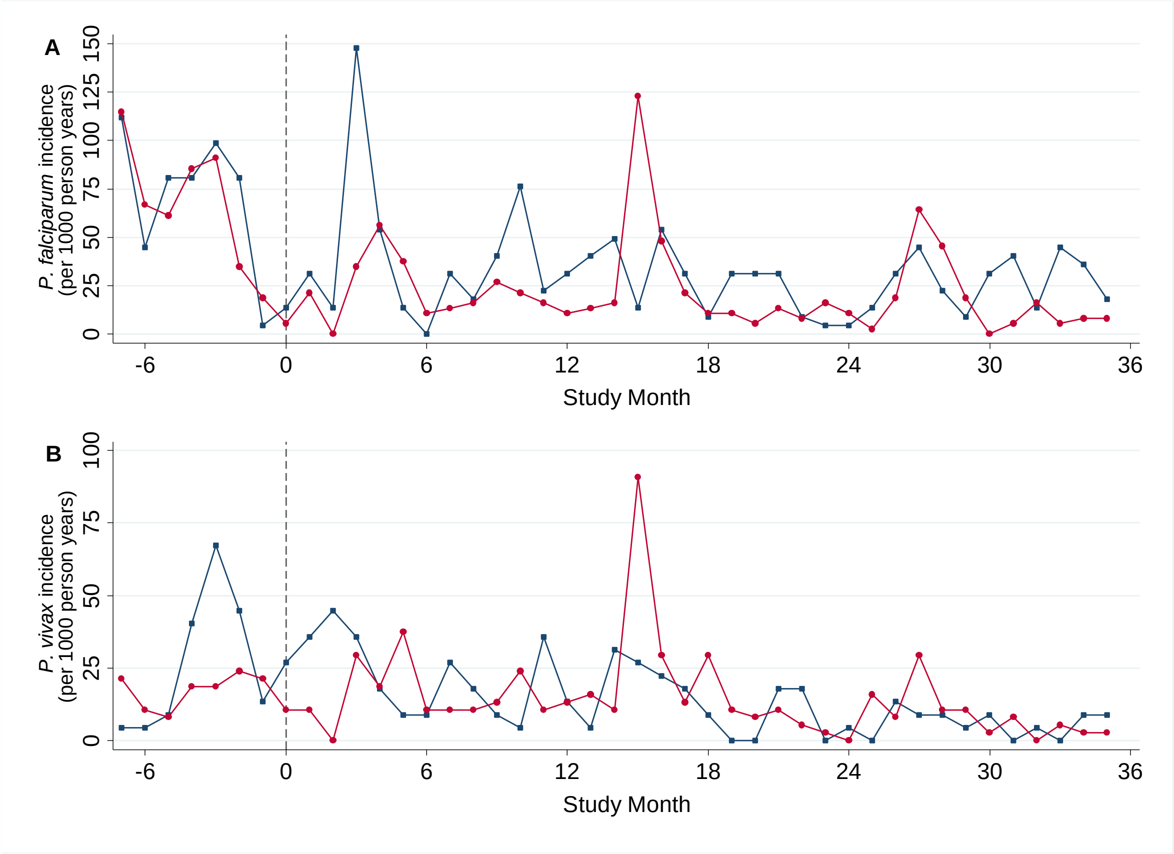

Supplementary Figure 3. Monthly (A) *P. falciparum* and (B) *P. vivax* incidence in intervention (red) and control arm (blue) in study pairs 3-8. Study pairs 3 through 8 all had operational CHWs from study month -7.

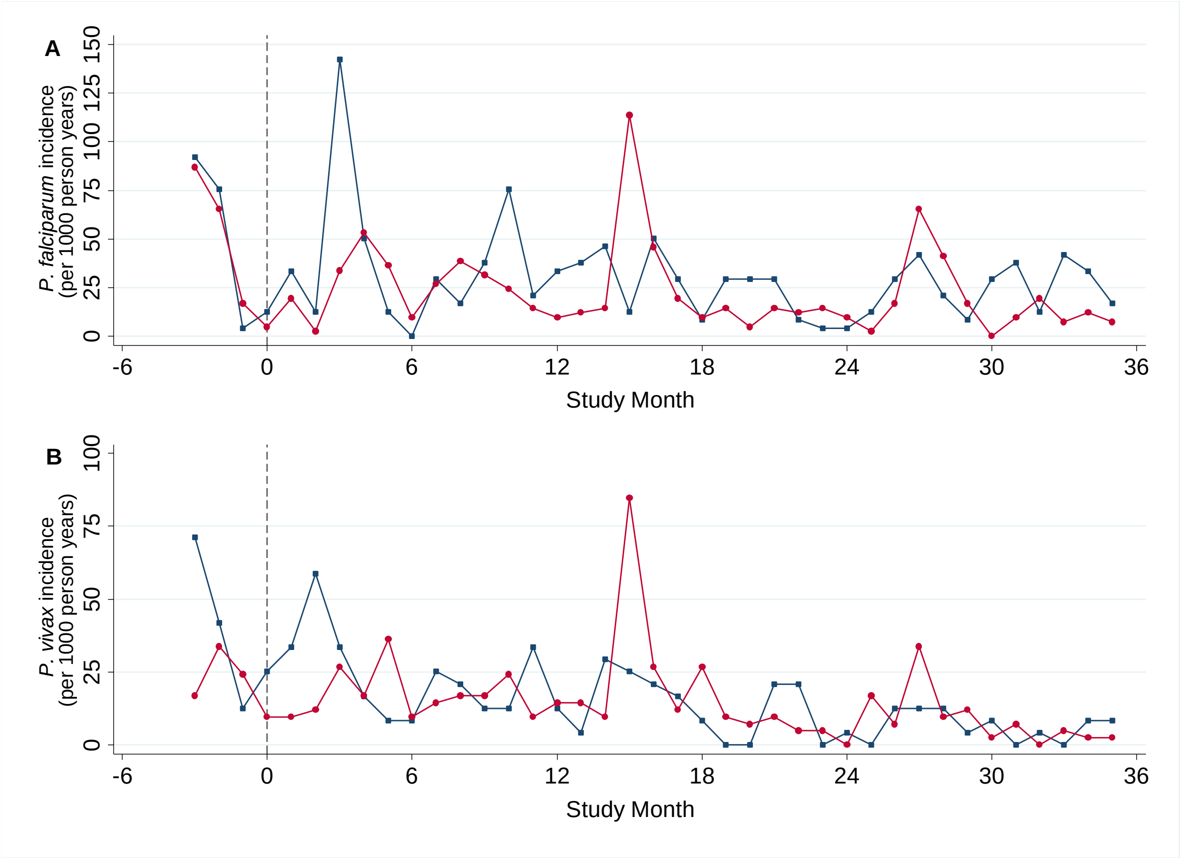

Supplementary Figure 4. Monthly (A) *P. falciparum* and (B) *P. vivax* incidence in intervention (red) and control arm (blue) in study pairs 2-8. Study pairs 2 through 8 all had operational CHWs from study month -3.

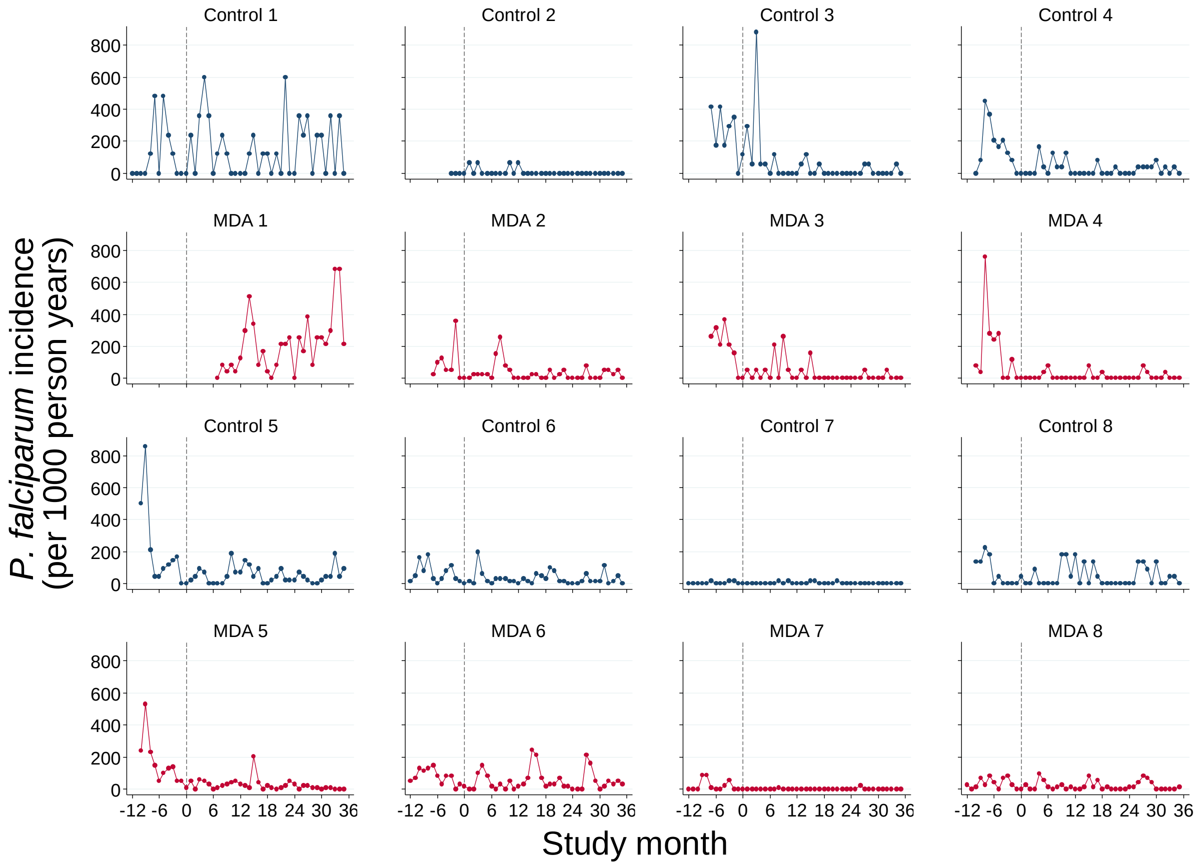

Supplementary Figure 5. Monthly *P. falciparum* incidence (per 1000 person years) in intervention (red) and control (blue) clusters.

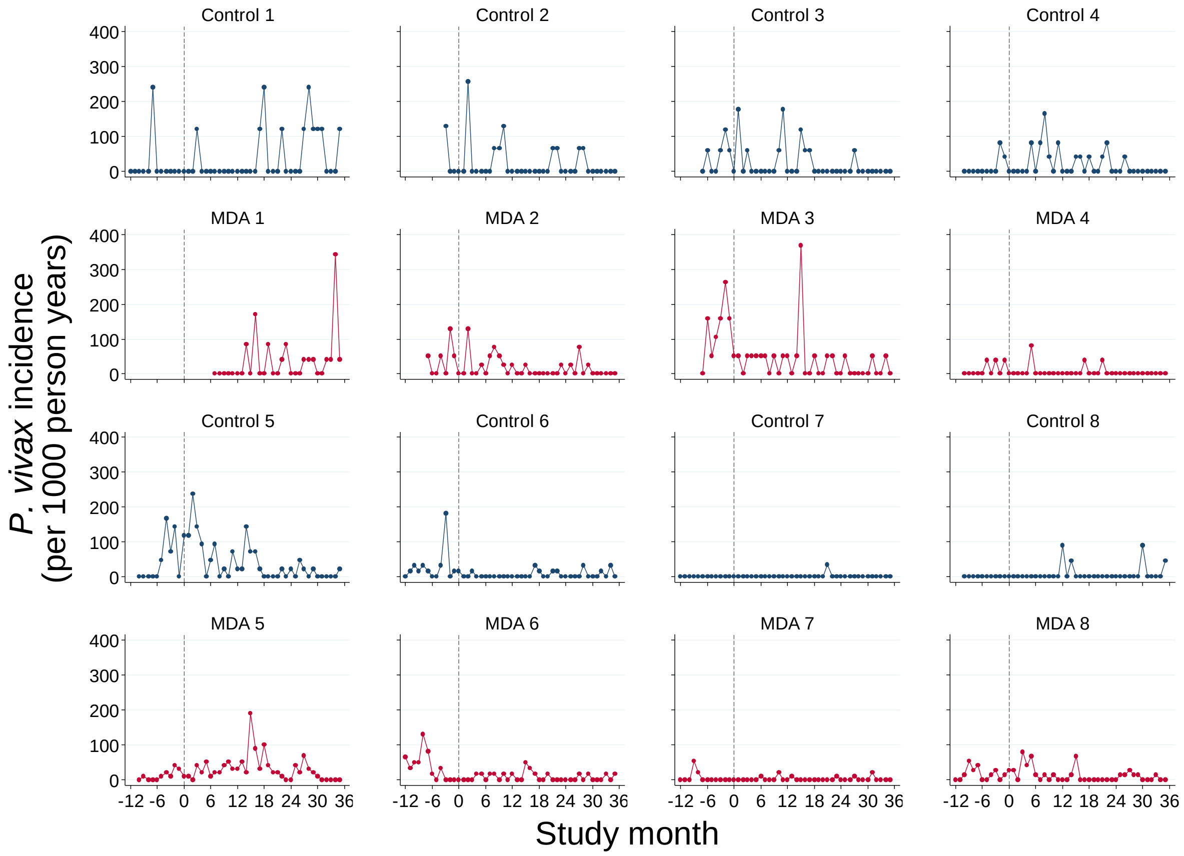

Supplementary Figure 6. Monthly *P. vivax* incidence (per 1000 person years) in intervention (red) and control (blue) clusters.
